# Correlated Noise Reduction in a CMOS ECG Amplifier: Opportunities and Limitations

**DOI:** 10.3390/s26185904

**Published:** 2026-09-17

**Authors:** Riccardo Olivieri, Giorgio Tatangelo, Mehran Khanehbeygi, Gianluca Barile, Leonardo Pantoli, Vincenzo Stornelli, Giuseppe Ferri

**Affiliations:** Department of Industrial and Information Engineering and Economics, University of L’Aquila, 67100 L’Aquila, Italy; giorgio.tatangelo@graduate.univaq.it (G.T.); mehran.khanehbeygi@graduate.univaq.it (M.K.); gianluca.barile@univaq.it (G.B.); leonardo.pantoli@univaq.it (L.P.); vincenzo.stornelli@univaq.it (V.S.); giuseppe.ferri@univaq.it (G.F.)

**Keywords:** biomedical signal acquisition, correlated noise reduction, ECG front-end, low-noise amplifier

## Abstract

Low-noise front-end amplifiers are essential for the acquisition of weak biopotential signals, such as electrocardiograms (ECGs), where the noise introduced by the first amplification stage directly limits the overall signal quality. While correlated noise-cancellation techniques have been extensively investigated for radio-frequency (RF) low-noise amplifiers, their application to low-frequency biomedical interfaces remains largely unexplored because of the different dominant noise mechanisms. This work investigates the applicability of an RF-inspired correlated noise-cancellation methodology to a low-frequency CMOS biomedical preamplifier core intended for ECG front-end applications. The proposed architecture extends a conventional resistive-feedback common-source amplifier by introducing an auxiliary feedforward path designed to attenuate the internally generated correlated noise components without significantly affecting the useful signal. The amplifier was designed in a 150 nm CMOS process and evaluated through AC, noise, Monte Carlo, PVT and time-domain analyses. Compared with the reference single-stage implementation, the proposed architecture achieves approximately a 20% reduction in integrated input-referred noise and a 14% reduction in integrated output-referred noise while marginally improving the voltage gain. Statistical analyses confirm that the proposed technique maintains its effectiveness under process, voltage, temperature, and mismatch variations. Time-domain evaluations with representative ECG waveforms further validate the methodology at the amplifier-core level and indicate its potential integration into complete low-noise biomedical analog front-ends.

## 1. Introduction

Biomedical sensing systems rely on the accurate acquisition of low-amplitude biopotential signals such as electrocardiogram (ECG), electromyogram (EMG), and electroencephalogram (EEG). These signals typically exhibit amplitudes ranging from a few microvolts to several millivolts and occupy relatively low-frequency bands, making them highly vulnerable to noise, interference, and signal distortion. In clinical and wearable monitoring applications, preserving signal integrity is essential, as any degradation can directly impact diagnostic accuracy, long-term trend analysis, and patient safety.

In a biopotential acquisition chain, the electronic analog interface plays a decisive role in determining the overall signal quality [1,2,3]. In particular, the early signal-conditioning chain includes a preamplifier, a low-noise amplifier (LNA), filtering stages, and a variable-gain amplifier (VGA). The first active block following the sensing electrodes typically dominates the system noise performance. Any noise added at this stage is directly referred to the input and cannot be effectively removed by subsequent analog filtering or digital signal processing without simultaneously degrading the desired signal. As a result, minimizing the noise contribution of the front-end amplifier is a fundamental requirement for biomedical sensor interfaces.

The challenge of low-noise amplification is further intensified by the stringent power and area constraints imposed by modern wearable and implantable devices. Long-term monitoring applications demand ultra-low power systems to extend battery lifetime or enable energy harvesting, while maintaining sufficient robustness against interference sources such as power-line coupling, motion artifacts, and electrode impedance variations. These constraints impose severe trade-offs among noise performance, power efficiency, and circuit complexity [4,5].

Conventional approaches to low-noise biopotential amplification typically rely on increasing the transconductance of the input devices, employing large passive components, or adopting offset and flicker-noise reduction techniques such as chopper stabilization and auto-zeroing [6,7]. While these methods can effectively reduce low-frequency noise, they often introduce additional drawbacks, including increased power consumption, chopping ripple, intermodulation artifacts, reduced dynamic range, or limited tolerance to large common-mode and transient disturbances. Moreover, improving noise performance by only purely passive or bias-current scaling becomes increasingly inefficient in deeply scaled CMOS technologies.

An alternative strategy to address these limitations is based on active noise cancellation within the amplifier itself. Rather than solely minimizing noise generation through device sizing or biasing, noise-reduction techniques exploit the correlation between internal noise sources and circuit nodes to suppress the contribution of dominant noise components at the output. By selectively canceling amplifier-generated noise while preserving the desired signal, such approaches offer the potential to relax the fundamental noise–power trade-off and achieve improved noise efficiency at low bias currents.

Noise-reduction principles have been extensively investigated in wideband and RF LNAs, where they have proven effective in reducing noise contributions without relying on high-Q passive components and using particular noise propagation paths without degrading the signal [8,9,10,11]. However, their application to low-frequency biomedical front-end amplifiers remains relatively unexplored, despite the strong relevance of noise efficiency and robustness in this domain. Adapting these techniques to biopotential acquisition introduces additional challenges, including stringent low-frequency noise requirements, stability with electrode impedance variations, and compatibility with low-voltage, low-power operation.

Motivated by these considerations, this work proposes a correlated noise-reduction methodology applied to a low-noise CMOS preamplifier core for ECG-oriented biomedical front-end applications. The goal is not to implement a complete ECG analog front-end, but to reduce the noise generated by the amplification core itself. Electrode interfacing, impedance adaptation, common-mode rejection, offset management, and complete filtering are outside the scope of this paper. To the best of the authors’ knowledge, this is among the first attempts to adapt RF-derived correlated noise-cancellation principles to a low-frequency CMOS biomedical preamplifier operating in a flicker-noise-dominated regime.

The remainder of this paper is organized as follows: Section 2 summarizes the dominant noise mechanisms in biomedical front-end amplifiers and introduces the correlated noise-reduction principle underlying the proposed approach. Section 3 introduces the proposed methodology and the amplifier architecture and describes the active noise reduction principle. Section 4 presents circuit-level analysis and design considerations, as well as simulation results. Finally, Section 5 concludes the paper and outlines future research directions.

## 2. Noise Reduction Techniques

**Figure 1:**
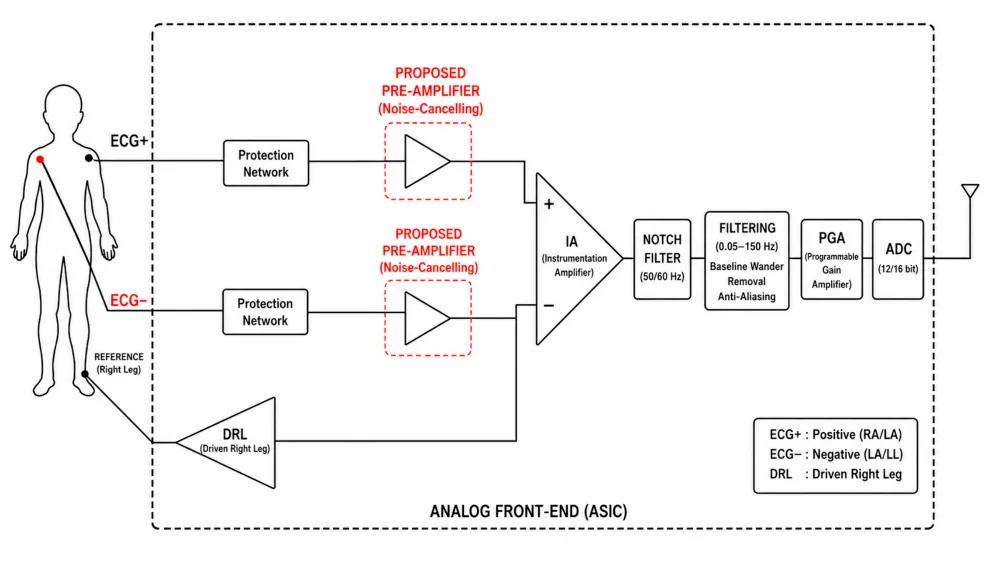
Possible system-level integration of the proposed noise-cancelling preamplifier core within a complete ECG analog front-end.

In typical ECG and biosignal acquisition chains, the preamplifier constitutes the first active block following the sensing electrodes, as shown in Figure 1. Therefore, its noise contribution is directly referred to the input and largely determines the total input-referred noise of the system. According to Friis’ noise relation, the noise contribution of the first active stage plays a dominant role in determining the overall input-referred noise, since the contributions of subsequent stages are reduced by the gain of the preceding stages [12,13]. Therefore, the design of low-noise front-end amplifiers remains a fundamental challenge for biomedical sensor interfaces.

The dominant noise sources in CMOS biomedical front-ends include the thermal and flicker noise of the input transistors, the thermal noise associated with resistive elements, and noise contributions given by the biasing networks [14].

**Figure 2:**
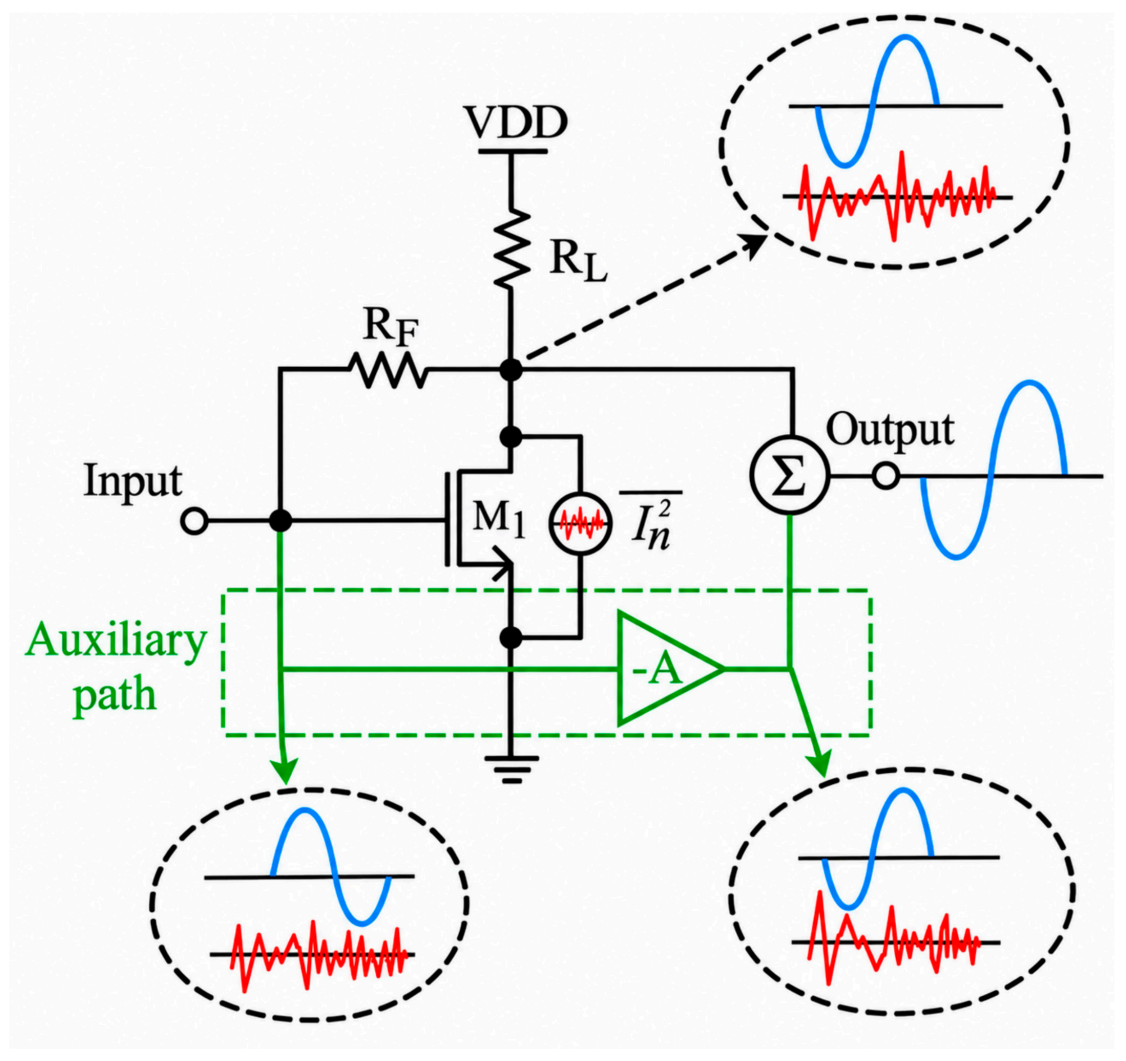
Noise-reduction concept applied to a common-source topology.

Figure 2 illustrates the operating principle of the noise-reduction mechanism. The main input transistor (M_1_) generates both the desired signal and intrinsic noise components. While the signal components appear with opposite polarity at the gate and drain of the transistor, the noise components observed at nodes $V_{a}$ and $V_{b}$ originate predominantly from the same device-noise source associated with M_1_ and are therefore expected to exhibit a significant degree of correlation. The actual magnitude and phase relationship between the two node-noise components is affected by the feedback network, finite device output conductances, and parasitic elements. Consequently, the effectiveness of the proposed cancellation mechanism depends on the degree of correlation preserved over the frequency range of interest. By appropriately sensing these correlated noise voltages at two nodes with identical polarity and routing them through two separate signal paths, they can be recombined at the output with opposite polarities. As a result, the noise components undergo destructive cancellation, whereas the desired signal components are constructively combined.

This feedforward noise-cancellation principle has been employed in wideband and RF LNAs, particularly in common-gate–common-source (CG–CS) and resistive-feedback architectures, where it effectively relaxes the fundamental trade-off between input matching and noise figure. Motivated by these advantages, this work adapts the underlying idea of the noise-reduction mechanism to the stringent constraints of biomedical applications, without seeking to replicate the exact cancellation condition of conventional RF implementations.

Chopper stabilization and auto-zeroing are commonly used in biomedical front-ends to reduce offset and flicker noise, but they may introduce ripple, modulation artifacts, additional feedback loops, and settling constraints [15,16,17,18]. In parallel, DRL feedback is typically employed to attenuate common-mode interference, especially power-line coupling; however, its effectiveness is limited by loop stability, electrode impedance, and safety constraints, and it does not reduce the intrinsic noise generated inside the amplifier core [19]. Therefore, the proposed methodology targets a complementary limitation: it aims to reduce the amplifier-generated noise at its source by exploiting the correlation between internal noise components and recombining them destructively at the output.

## 3. Proposed Architecture and Noise Analysis

### 3.1. Proposed Architecture

**Figure 3:**
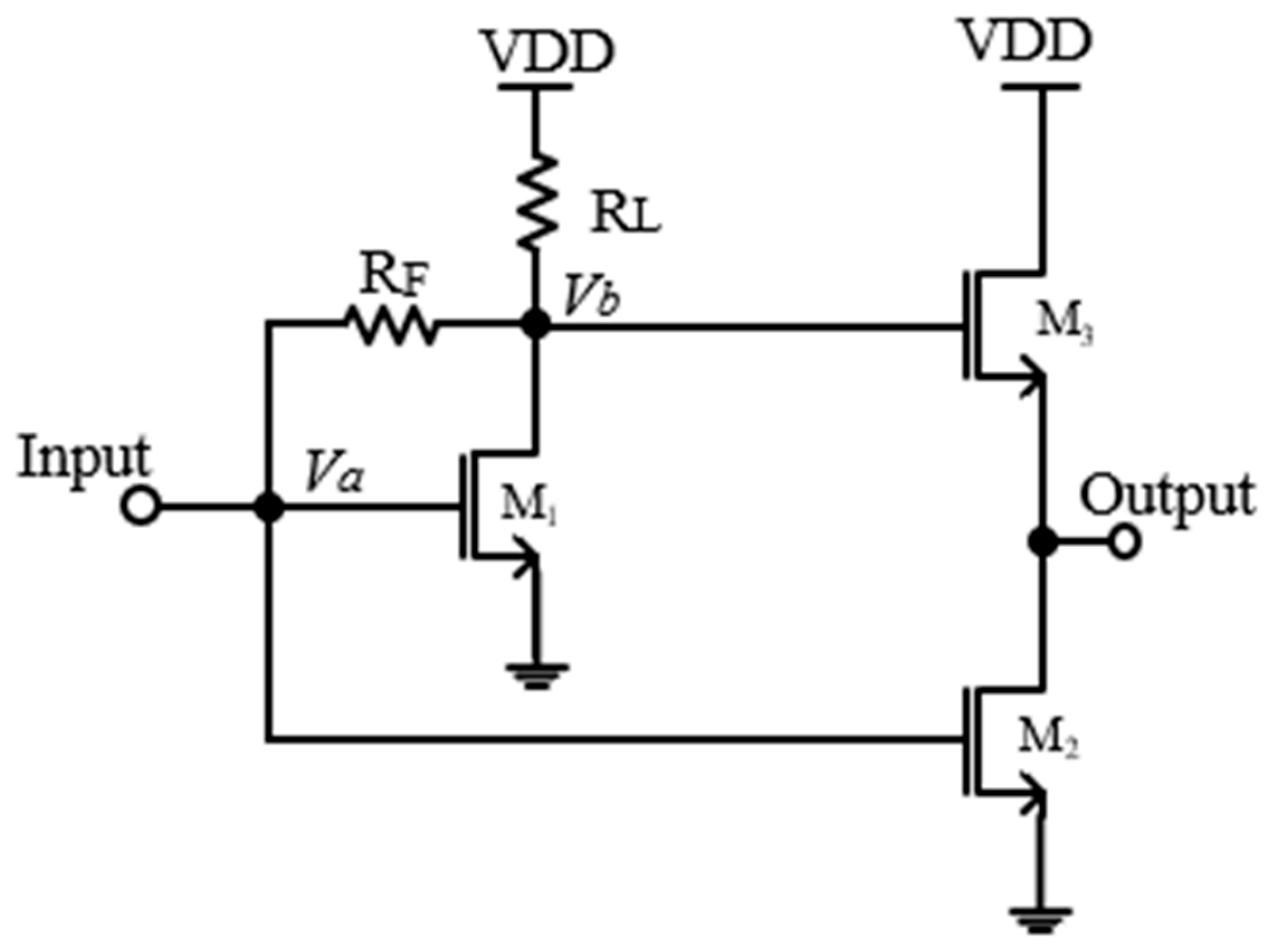
Adopted topology implementing the noise-reduction mechanism.

To investigate the applicability of noise-reduction techniques in low-frequency biomedical front-end amplifiers, two circuit configurations were designed and comparatively analyzed. The first topology consists of a conventional single-stage common-source preamplifier with resistive shunt feedback, which serves as the reference architecture. The second topology extends the same structure by introducing an auxiliary noise-cancelling path, yielding a two-stage front-end amplifier. Figure 3 illustrates the proposed noise-cancelling architecture. In the reference configuration, transistor M_1_ operates as the main amplifying device. A resistive feedback network consisting of *R_F_* establishes the DC operating point while simultaneously influencing the small-signal gain and input impedance behavior. The load resistor *R_L_* converts the drain current variations into an output voltage.

The proposed architecture retains the same input stage and introduces two additional transistors, M_2_ and M_3_, to implement the auxiliary noise-cancelling path. The operating principle is based on the correlation properties of the noise components generated by the main transistor M_1_. While the useful signal components appearing at different internal nodes exhibit opposite polarity, the noise components generated by the channel of M_1_ remain correlated and appear with identical polarity. By appropriately processing these correlated noise components through two separate signal paths and recombining them at the output, a partial suppression of the internally generated noise contribution can be achieved. The reference single-stage amplifier employs M_1_ with dimensions W/L = 9 μm/150 nm and number of fingers (nf) = 20. The feedback resistor is set to *R_F_* = 100 kΩ, while the load resistor is *R_L_* = 200 kΩ. The output of the proposed architecture is taken from the drain node shared by M_2_ and M_3_, where the contributions of the main amplification path and the auxiliary reduction path are recombined. Unlike conventional RF implementations, the auxiliary path is not intended to achieve complete noise cancellation, but rather to investigate the achievable noise reduction under the operating conditions typical of low-frequency biomedical interfaces. The estimated area of the circuit, excluding the large resistive elements and routing, is about 15 μm × 20 μm.

### 3.2. Small-Signal Gain Analysis

Applying the standard small-signal MOSFET model to the reference single-stage amplifier and accounting for the resistive feedback network, the voltage gain can be approximated as

(1) $$Av=\frac{R_{L}\;(1-g_{m}R_{F})}{R_{L}+R_{F}+R_{s}(1+g_{m}R_{L})}$$

where *g_m_* denotes the transconductance of transistor M_1_, *R_L_* is the load resistance, *R_F_* is the feedback resistance, and *R_S_* represents the equivalent source resistance seen at the input.

Equation (1) shows that the voltage gain of the reference common-source amplifier depends jointly on $g_{m}$, $R_{F}$, $R_{L}$, and $R_{S}$. Therefore, increasing $R_{F}$ or $g_{m}$ does not provide an independent gain enhancement, since both parameters also affect the feedback-induced loading and the equivalent input impedance. In the proposed noise-cancelling topology, the auxiliary branch introduces an additional transconductance-dependent path at the output node. As a result, the overall small-signal response can be interpreted, in a first-order approximation, as the superposition of the main amplification path and the auxiliary branch contribution. The circuit must therefore be designed as a compromise between gain enhancement, noise reduction, current consumption, and operating-point stability.

### 3.3. First-Order Noise Analysis

The power spectral density of the MOSFET thermal channel noise component can be expressed as:

(2) $$\overset{\_}{i_{n,M_{1}}^{2}}\left(f\right)=4kT\gamma \;g_{m1}\;\;[A^{2}/\mathrm{Hz}]$$

(3) $$\;\overset{\_}{i_{n,R_{L}}^{2}}\left(f\right)=\frac{4kT}{R_{L}}\;[A^{2}/\mathrm{Hz}]$$

where k, T, γ represent the Boltzmann constant, the absolute temperature and the thermal noise factor, respectively. Assuming statistically independent noise sources and neglecting second-order effects such as body-induced noise and parasitic capacitances, the effective conductance at node *V_b_* can be approximated as

(4) $$G_{b}≅g_{ds1}+\frac{1}{R_{L}}+\frac{1}{R_{F}+R_{S}}+\alpha g_{m1}$$

with

(5) $$\alpha ≅\frac{R_{S}}{R_{S}+R_{F}},\;V_{a}=\alpha V_{b}$$

where *g_ds_*_1_ is the output conductance of M_1_, while α represents the attenuation factor between nodes *V_b_* and *V_a_* due to the feedback network.

Under these assumptions, the output noise contribution generated by the auxiliary noise-reduction path can be expressed as

(6) $$v_{\mathrm{out}}=-R_{\mathrm{out}}\;i_{\mathrm{out}}=-R_{\mathrm{out}}\left(\alpha g_{m2}-g_{m3}\right)V_{b}\;$$

where *R_out_* is the equivalent output resistance, and *g_m_*_2_ and *g_m_*_3_ are the transconductances of the auxiliary transistors M_2_ and M_3_, respectively. Equation (6) shows that the noise contribution at the output depends on the difference between the two transconductance-weighted paths. Therefore, by properly selecting the auxiliary transconductances, the internally generated noise contribution can be partially recombined with opposite polarity at the output.

The corresponding noise transfer function from the channel noise current of M_1_ to the output voltage can be approximated as:

(7) $$T_{n,M1}\left(j\omega \right)\;=\;\frac{v_{\mathrm{out}}}{i_{n1}}=\frac{R_{\mathrm{out}}\left(\alpha g_{m2}-g_{m3}\right)}{G_{b}}\;\;$$

Equation (7) indicates that the contribution of the noise generated by M_1_ can be shaped through the transconductance ratio of the auxiliary branch.

To explicitly quantify the correlation underlying the proposed mechanism, the transfer of the M_1_-generated noise toward nodes $V_{a}$ and $V_{b}$ can be separately expressed as:

(8) $$H_{n,a}\left(j\omega \right)=\frac{v_{n,a}}{i_{n,M_{1}}},\;\;\;\;\;\;\;\;\;H_{n,b}\left(j\omega \right)=\frac{v_{n,b}}{i_{n,M_{1}}}\;$$

Accordingly, the corresponding auto- and cross-spectral densities are

(9) $$S_{aa}={\left|H_{n,a}\right|}^{2}S_{M_{1}},\;\;S_{bb}={\left|H_{n,b}\right|}^{2}S_{M_{1}},\;S_{ab}=H_{n,a}H_{n,b}^{*}S_{M_{1}}$$

where $S_{M1}$ denotes the power spectral density of the noise generated by M_1_. The magnitude-squared coherence between the two noise components can therefore be defined as:

(10) $$\gamma_{ab}^{2}\left(f\right)=\frac{{\left|S_{ab}\left(f\right)\right|}^{2}}{S_{aa}\left(f\right)S_{bb}(f)}\;$$

Under the first-order relation $V_{a}=\alpha V_{b\;}$ given in (5), the two node-noise components originate from the same stochastic source and are related by $H_{n,a}≃\alpha H_{n,b}$. Therefore, the M1-generated components are ideally fully coherent, i.e., $\gamma_{ab,M1}^{2}=1$, although their amplitudes differ by the factor α. Importantly, unity coherence does not imply equal noise amplitudes, but indicates that the two node voltages represent deterministic transformations of the same M_1_-generated noise source. In the complete transistor-level circuit, finite output conductances, parasitic elements, and additional independent device-noise sources reduce the total-node coherence from its ideal value.

It is important to emphasize that the correlated-noise reduction principle is not intrinsically restricted to thermal noise. In RF low-noise amplifiers, the method has historically been applied mainly in frequency ranges where thermal noise represents the dominant device-noise mechanism. However, the underlying cancellation condition depends on the correlation between noise components generated by the same stochastic source and transferred through deterministic circuit paths, rather than on the specific spectral shape of that source.

The same argument therefore applies to the flicker-noise component generated by M_1_. Denoting the flicker-noise source of M_1_ by $n_{M1,1/f}$, the corresponding node voltages can be written as:

(11) $${\;v}_{n,a}^{1/f\;}=H_{n,a\;}\left(j\omega \right)n_{M_{1},1/f\;},\;{\;\;\;\;\;\;\;\;\;v}_{n,b}^{1/f\;}=H_{n,b\;}\left(j\omega \right)n_{M_{1},1/f\;}$$

The associated cross-spectral density is consequently:

(12) $$S_{ab}^{\frac{1}{f}}\left(f\right)=H_{n,a\;}\left(j\omega \right)H_{n,b}^{*}\left(j\omega \right)S_{M_{1}}^{\frac{1}{f}}\left(f\right)\;$$

where $S_{M1}^{1/f}\left(f\right)\propto 1/f$. Since the same spectral term appears in the corresponding auto-spectral densities, it cancels when the normalized coherence is evaluated. Therefore, under the single-dominant-source assumption, the ideal coherence remains unity independently of whether the underlying noise spectrum is white or follows a 1/f dependence. The relevant requirement for cancellation is thus the preservation of the relative amplitude and phase relationship between the two propagated noise components.

This observation is particularly relevant to the present low-frequency operating regime, where the total amplifier noise is strongly dominated by the flicker contribution of M_1_. Consequently, a large fraction of the noise observed at $V_{a\;}$ and $V_{b}$ originates from the same device-noise process, preserving the correlation required by the auxiliary cancellation path. The proposed architecture should therefore be interpreted as an extension of the correlated-noise cancellation concept from the thermal-noise-dominated RF regime to a flicker-noise-dominated biomedical regime.

In the ideal case, the dominant noise contribution would be cancelled when the auxiliary path exactly compensates for the noise component transferred through the main path. This leads to the following approximate ideal cancellation condition:

(13) $$g_{m2}=\;g_{m3}(1+\frac{R_{F}}{R_{S}})$$

Equation (13) shows that complete cancellation requires a very large auxiliary transconductance when the feedback resistance *R_F_* is much larger than the source resistance *R_S_*. For the adopted design values, *R_F_* = 100 kΩ, *R_S_* = 50 Ω, and *g_m_*_3_ = 2.17 mS, the ideal value of *g_m_*_2_ would be:

(14) $$g_{m2,ideal\;\;}\approx 2.17\;mS\;\cdot \left(1+\frac{100\;k\Omega }{50\;\Omega }\right)\;\approx 4.3\;S$$

Such a high transconductance value would require a substantially larger auxiliary transistor and a significantly higher bias current. This would be incompatible with the target of the proposed biomedical front-end in terms of power and area, and would also modify the voltage gain, bandwidth, and operating point of the amplifier. For this reason, the auxiliary branch was intentionally designed to provide partial, rather than ideal, noise reduction, as anticipated in Section 1. In the implemented topology, transistors M_2_ and M_3_ are sized with W/L = 13.5 μm/150 nm and nf = 30, resulting in simulated transconductances of approximately 2.13 mS and 2.17 mS for *g_m_*_2_ and *g_m_*_3_, respectively. This sizing was selected to operate deliberately away from the ideal cancellation condition, providing a finite reduction of the dominant M_1_-generated noise while limiting the additional device size, current consumption, and parasitic loading introduced by the auxiliary branch.

To quantitatively assess the effectiveness of the auxiliary path at the adopted operating point, the noise transfer associated with M_1_ can be normalized with respect to the reference single-stage configuration. In the reference amplifier, the channel-noise current generated by M_1_ produces a voltage at node $V_{b}$, which also represents the amplifier output. Under the first-order assumptions introduced above, the corresponding noise transfer can therefore be approximated as:

(15) $$T_{n,M1}^{ref}\approx \frac{1}{G_{b}}.\;$$

Conversely, for the proposed architecture, the M_1_-generated noise is transferred to the output according to (7). Therefore, a normalized M_1_-noise transfer factor can be defined as:

(16) $$\eta_{M1}=\left|\frac{T_{n,M1}^{prop}}{T_{n,M1}^{ref}}\right|=R_{out}\left|\alpha g_{m2}-g_{m3}\right|.\;$$

The corresponding reduction of the RMS noise contribution generated by M_1_ can then be estimated as:

(17) $$NR_{M1}=\left(1-\eta_{M1}\right)\;\times 100\%.\;$$

For the adopted design values, $R_{S}=50\;\Omega$, $R_{F}=100\;$kΩ, $g_{m2}=2.13\;$mS, and $g_{m3}=2.17\;$mS, the attenuation factor is $\alpha =4.998\times 10^{-4}$. The simulated small-signal output resistance of the auxiliary branch is approximately $R_{out}=329\;\Omega$. Consequently,

(18) $$\eta_{M1}\approx 329\left|\left(4.998\times 10^{-4}\right)\left(2.13\times 10^{-3}\right)-2.17\times 10^{-3}\right|\approx 0.714,\;$$

corresponding to an expected reduction of approximately 28.6% in the RMS noise contribution generated by M_1_. Therefore, although the implemented value of $g_{m2}$ is approximately three orders of magnitude lower than the value required for ideal cancellation, the first-order model predicts a finite and significant attenuation of the dominant M_1_ noise contribution. The design should thus be interpreted as a partial noise-cancellation operating point rather than as an approximation of the ideal zero-noise condition.

This analytical prediction is consistent with the transistor-level noise-contribution analysis presented in Section 4.3, where the integrated contribution of M_1_ decreases from approximately 58.0 µVrms in the reference amplifier to 41.4 µVrms in the proposed architecture, corresponding to a reduction of approximately 28.6%. The smaller improvement observed in the total integrated noise is due to the additional noise contributions introduced by M_2_ and M_3_.

It should be noted that $R_{S}\;$ represents the equivalent source resistance adopted in the simplified small-signal and noise model, and it should not be directly interpreted as the electrode–skin impedance of a practical ECG interface. In real ECG acquisition systems, the electrode impedance is generally higher, frequency-dependent, and subject to large variations due to electrode type, skin preparation, motion, and contact conditions. As shown by Equation (13), the ideal cancellation condition is sensitive to $R_{S}$, since the required auxiliary transconductance decreases as $R_{S}\;$ increases. Therefore, the value obtained for $R_{S}=$ 50 Ω represents a conservative and controlled design condition rather than a universal ECG operating point. This further supports the choice of implementing only partial noise reduction instead of enforcing ideal cancellation, which would be strongly dependent on the actual source impedance. In a complete ECG front-end, the proposed amplification core should be embedded after the electrode protection and biasing network within a high-input-impedance differential architecture, so that the effective impedance seen by the noise-cancelling core can be adequately controlled.

The dependence of the ideal cancellation condition on the source resistance provides further insight into the expected behavior under higher-impedance source conditions. According to Equation (13), the auxiliary transconductance required for ideal cancellation decreases monotonically as $R_{S}$ increases. For the adopted values $R_{F}=100\;$kΩ and $g_{m3}=2.17\;$mS, the ideal value of $g_{m2}$ decreases from approximately 4.34 S at $R_{S}=50\;\Omega$ to 219 mS at 1 kΩ, 23.9 mS at 10 kΩ, 4.34 mS at 100 kΩ and 2.39 mS at 1 MΩ. Therefore, the implemented value $g_{m2}=$ 2.13 mS, which is intentionally far from the ideal cancellation condition at $R_{S}=$ 50 Ω, progressively approaches the ideal condition as the source resistance increases.

Within the adopted first-order model, the low value $R_{S}=$ 50 Ω can therefore be interpreted as a conservative limiting condition with respect to the cancellation requirement, since it maximizes the ratio $R_{F}/R_{S}\;$ and hence the auxiliary transconductance required for ideal noise suppression. In this sense, the 50 Ω assumption does not artificially favor the proposed technique; rather, it represents a worst-case-like condition for the cancellation mechanism. Higher source impedances, more representative of practical electrode-interface conditions, make the ideal cancellation requirement progressively less demanding.

It should nevertheless be emphasized that a practical ECG electrode exhibits a complex, frequency-dependent impedance rather than a purely resistive source impedance. Therefore, the present analysis should be interpreted as a first-order sensitivity assessment of the cancellation mechanism with respect to source resistance. A complete validation including a frequency-dependent electrode–skin impedance model is left to future system-level investigation.

A transistor-level parametric verification of the noise-reduction performance as a function of $R_{S}$ is provided in Section 4.5.

Although these values do not satisfy the ideal cancellation condition given in Equation (13), they allow a practical compromise between noise suppression and gain preservation. In particular, increasing *g_m_*_2_ and *g_m_*_3_ further would improve the theoretical noise reduction capability, but at the cost of increased current consumption, larger parasitic capacitances, and a possible degradation of the small-signal gain and bandwidth.

Therefore, the proposed topology does not aim to obtain a complete cancellation of the internally generated noise. Instead, it exploits the auxiliary path to achieve a controlled reduction of the dominant noise contribution while maintaining a suitable voltage gain. The effectiveness of this partial cancellation mechanism depends on the operating frequency regime and on the relative weight of flicker and thermal noise contributions, as discussed in the following simulation results.

## 4. Simulation Results and Discussion

### 4.1. Simulation Environment and Design Choices

**Figure 4:**
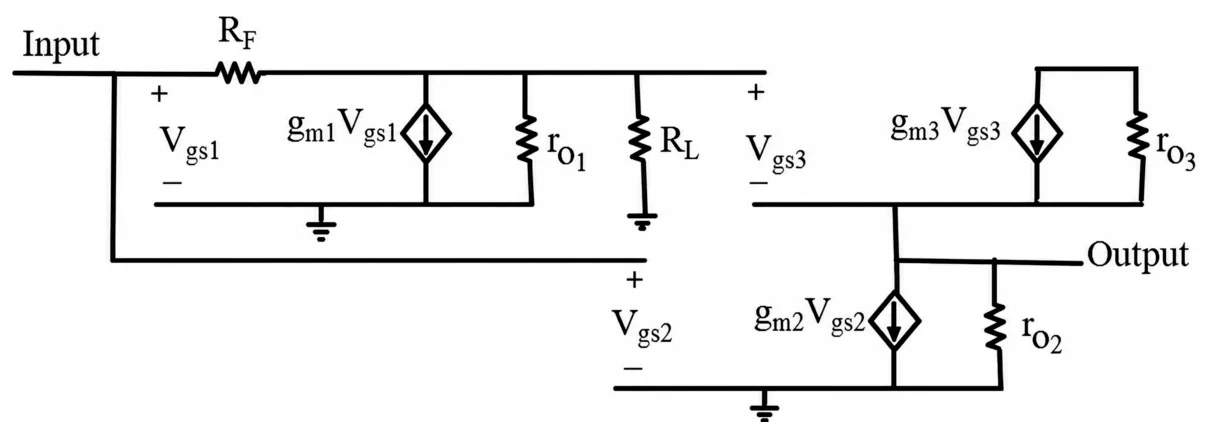
Small-signal equivalent circuit of the proposed noise-cancelling LNA.

To evaluate the applicability of the proposed noise-reduction architecture to low-frequency biomedical front-end amplifiers, both the conventional single-stage amplifier and the proposed two-stage topology were designed and simulated at transistor level using the LFoundry 150 nm standard CMOS technology. Unless otherwise specified, all simulations were performed under typical process conditions at room temperature. Both configurations share the same input stage and were designed using the same M_1_ device sizing and nominal input-stage supply conditions to ensure a fair comparison in terms of gain and noise performance. The reference amplifier consists of a common-source stage. The proposed architecture, whose small-signal equivalent circuit is depicted in Figure 4, preserves the same input stage while introducing two auxiliary transistors, M_2_ and M_3,_ which implement the correlated noise-reduction path described in Section 3.3.

**Figure 5:**
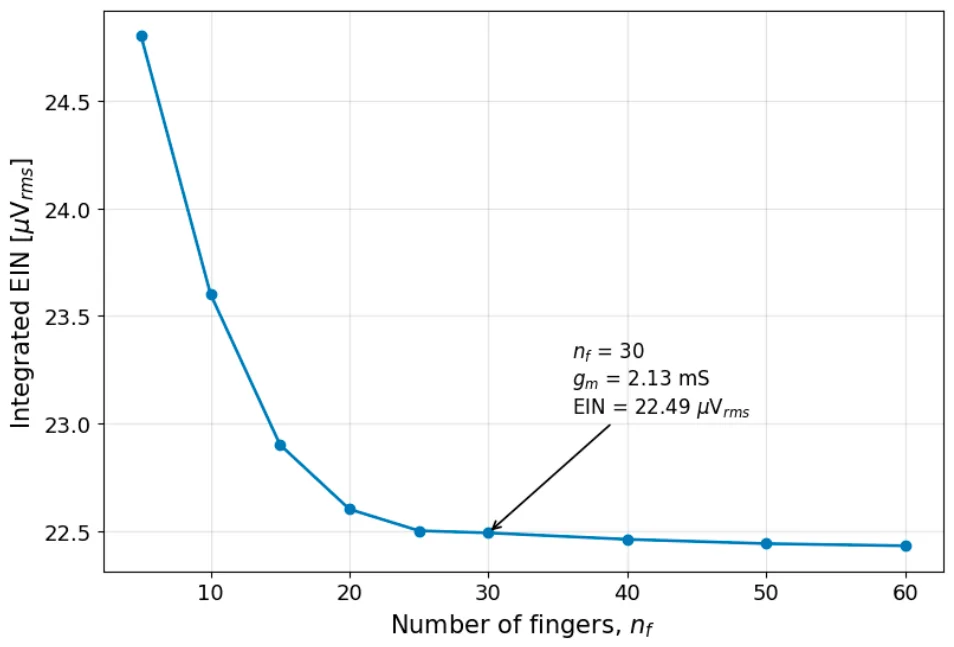
Integrated equivalent input noise as a function of the number of fingers nf of the auxiliary transistors M_2_ and M_3_.

A parametric sweep of the number of fingers of M_2_ and M_3_ was performed while keeping the two auxiliary devices identically sized. Figure 5 reports the integrated equivalent input noise as a function of nf. Increasing nf initially reduces the integrated noise because the larger auxiliary transconductance improves the attenuation of the M_1_-generated noise contribution. However, beyond approximately nf = 30, the improvement becomes progressively smaller, since the increased transconductance and device area of M_2_ and M_3_ also increase their intrinsic noise contribution and parasitic loading. The selected value nf = 30 therefore lies close to the onset of the saturation region of the noise-reduction curve and represents a practical compromise between cancellation effectiveness, auxiliary-device self-noise, and circuit complexity. Increasing nf beyond the selected value produces only a marginal additional reduction in integrated noise while increasing current consumption and parasitic capacitance.

This behavior is consistent with two simultaneous effects: any movement of *g_m_*_2_ toward the ideal value marginally improves the reduction, but the proportional increase of the self-noise of M_2_ and M_3_, which scales with their own transconductance, exceeds the gain in reduction in this region of the design space. The two effects compensate to a degree that depends on the band of integration, and the net outcome differs between the flicker-dominated and the thermal-dominated regions of the spectrum. The DC operating points of the reference and proposed configurations were extracted from the transistor-level simulations and are summarized here. In the reference topology, transistor M_1_ operates with a drain current of 2.164 µA, a transconductance of 55.67 µS, and an output conductance of 2.5 µS. In the proposed topology, the loading introduced by the auxiliary branch slightly modifies the operating point of M_1_, reducing its drain current to 1.799 µA and its transconductance to 46.72 µS. The auxiliary transistors M_2_ and M_3_ operate at substantially higher current levels, 160.6 µA each, and provide transconductances of 2.13 mS and 2.17 mS, respectively. The input stage M_1_ is supplied from the nominal 460 mV rail, whereas the M_2_–M_3_ auxiliary branch is biased from a dedicated 400 mV supply. The current consumption of the two configurations was also evaluated in order to quantify the cost associated with the auxiliary noise-reduction branch. In the reference amplifier, transistor M_1_ draws 2.164 µA from the 460 mV supply, corresponding to a DC power consumption of 0.995 µW. In the proposed topology, M_1_ draws 1.799 µA from the same 460 mV supply, corresponding to 0.828 µW. Transistors M_2_ and M_3_ form a series auxiliary branch biased from a dedicated 400 mV supply and therefore carry the same branch current of 160.6 µA. The corresponding auxiliary-branch power consumption is 64.24 µW. The total DC power consumption of the proposed amplifier core is therefore 65.07 µW, compared with approximately 1.00 µW for the reference topology.

The noise–current trade-off was further quantified through the conventional Noise Efficiency Factor (NEF), evaluated using the integrated input-referred noise over the selected 0.01 Hz–1 kHz system-level evaluation bandwidth. The reported NEF values therefore refer to this application-oriented bandwidth and should not be interpreted as values obtained by integrating the noise over the intrinsic hundreds-of-megahertz bandwidth of the uncompensated amplifier core. The reference amplifier exhibits an NEF of 50.3, whereas the proposed topology yields an NEF of 349.4. Therefore, although the auxiliary branch reduces the dominant M_1_-generated noise contribution, the present implementation does not improve noise efficiency because the reduction is obtained at the expense of a substantially larger bias current. These results further confirm that the proposed circuit should be interpreted as a proof-of-concept implementation of the correlated-noise-reduction mechanism rather than as an optimized ultra-low-power biomedical amplifier.

Therefore, the proposed topology should not be interpreted as an improvement in noise–power efficiency, but rather as a proof-of-concept demonstration of correlated noise reduction in a flicker-noise-dominated biomedical amplifier.

The DC output level is established by the biasing of the auxiliary M_2_–M_3_ branch. The transistor-level DC operating-point simulation yields a nominal output voltage of 171.3 mV.

The extracted DC operating points further characterize the bias conditions of the auxiliary devices. In particular, M_2_ exhibits a transconductance of 2.13 mS and an output conductance of 203 µS, with |V_GS_| = 628.7 mV, |V_DS_| = 171.3 mV, |V_TH_| = 540.8 mV, and V_DSAT_ = 108.4 mV. M_3_ operates under comparable conditions, with g_m_ = 2.17 mS and g_ds_ = 232 µS, while the corresponding voltage quantities are |V_GS_| = 600 mV, |V_DS_| = 228.7 mV, |V_TH_| = 511.4 mV and V_DSAT_ = 108.9 mV. The close transconductance values of the two auxiliary devices are consistent with their role in implementing the two correlated signal paths required by the partial noise-reduction mechanism.

Operation in weak inversion is particularly attractive for low-power biomedical front-end amplifiers because it maximizes the transconductance efficiency *gm/I_D_*. In this regime, *gm/I_D_* approaches its theoretical upper bound, approximately given by 1/(nV_T_), where *n* is the subthreshold slope factor and V_T_ = kT/q is the thermal voltage. Therefore, for a given bias condition, weak-inversion operation allows a relatively high transconductance to be achieved, which is beneficial for low-noise operation in low-power biomedical front-end circuits. Nevertheless, this efficiency is not obtained without cost. Weak-inversion operation limits the achievable transit frequency, and hence the speed and bandwidth of the stage. It also increases the sensitivity to process and mismatch variations, since the drain current depends exponentially on V_GS_–V_TH_; and, because the high *gm/I_D_* reduces the input-referred thermal noise floor, it raises the 1/f corner frequency, so that flicker noise dominates over a wider portion of the spectrum. For the target application these trade-offs are acceptable: the ECG bandwidth lies orders of magnitude below the bandwidth limit of the stage, the robustness against process and mismatch variations is explicitly verified in Section 4.4, and the resulting flicker-dominated regime is precisely the operating condition that the proposed correlated-noise attenuation method is intended to address.

However, biasing the transistor too deeply in weak inversion reduces the absolute value of *gm*, eventually limiting the achievable voltage gain. This behavior was verified through simulations, which showed that an excessive reduction of the bias current leads to a measurable degradation of the amplifier gain, despite the higher transconductance efficiency. The selected operating point therefore represents a good compromise between sufficient voltage gain and low-noise performance.

The auxiliary transistors were intentionally sized to provide only a partial fulfillment of the ideal noise-reduction condition derived in Section 3.3. Although larger auxiliary transconductances would lead to a closer approximation to the ideal cancellation condition, they would also require higher bias currents and larger device dimensions. This would increase power consumption, introduce additional parasitic capacitances, and significantly alter the gain and bandwidth of the amplifier. Consequently, the adopted sizing of M_2_ and M_3_ represents a deliberate trade-off between noise suppression and gain preservation, consistent with the requirements of biomedical front-end interfaces.

### 4.2. Small-Signal Performance

The small-signal AC response of both amplifier configurations was evaluated to quantify the effect of the auxiliary noise-reduction path on the voltage gain and frequency response.

**Figure 6:**
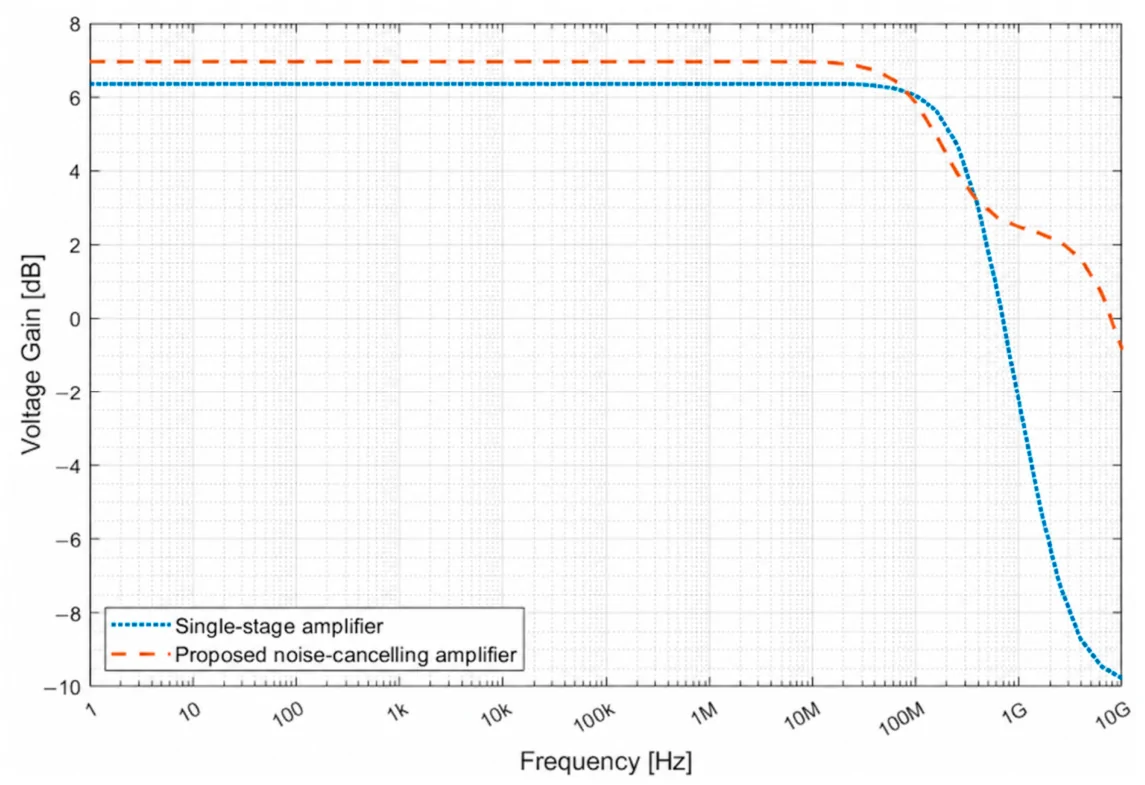
AC voltage gain of the reference single-stage amplifier and the proposed noise-cancelling amplifier.

The reference single-stage amplifier exhibits a simulated mid-band gain of approximately 6.34 dB, remaining substantially constant up to a −3 dB bandwidth of 398.11 MHz. Introducing the auxiliary stage increases the overall gain to approximately 6.95 dB, with a bandwidth of 251.89 MHz, as illustrated in Figure 6.

The observed gain improvement confirms that the auxiliary branch contributes not only to the correlated-noise suppression mechanism but also provides an additional signal path that constructively reinforces the desired output signal. Consequently, the proposed architecture achieves a simultaneous gain enhancement and partial noise reduction.

The secondary high-frequency plateau observed in the proposed response results from the interaction between the main and auxiliary feedforward paths, whose different parasitic poles introduce a pole–zero interaction before the final roll-off. Since the auxiliary branch is primarily feedforward, it does not introduce an additional global feedback loop. It should be emphasized that the proposed circuit is not intended to provide the complete voltage gain or frequency selectivity of an ECG analog front-end. Rather, it represents a low-noise preamplifier core used to investigate the effect of the proposed correlated-noise-reduction mechanism independently of the subsequent gain and filtering stages. In the system-level architecture illustrated in Figure 1, the overall gain is distributed among the preamplifier core, the subsequent instrumentation amplifier, and the programmable-gain stage. Therefore, the gain reported here should not be interpreted as the total ECG front-end gain.

Similarly, the −3 dB bandwidths of 398.11 MHz and 251.89 MHz represent the intrinsic transistor-level bandwidths of the two uncompensated amplification cores and are not intended to define the ECG signal bandwidth. No explicit low-frequency band-limiting network was included in the investigated core in order to isolate the effect of the auxiliary noise-reduction path. In a complete implementation, the useful bandwidth would be restricted by the dedicated filtering stages shown in Figure 1 to the frequency range required for ECG acquisition. Consequently, the large intrinsic bandwidth reported here does not imply that broadband signals would be passed throughout the complete ECG acquisition chain.

The very large intrinsic bandwidths reported for both cores should therefore not be interpreted as a design target for ECG acquisition, but rather as a consequence of evaluating the uncompensated preamplifier structures without an internal bandwidth-limiting network. This choice was intentionally retained in the present proof-of-concept study to avoid introducing additional poles or filtering elements that could modify the relative noise-transfer functions of the main and auxiliary paths and thereby obscure the specific effect of the correlated noise-reduction mechanism. From a complete biomedical front-end perspective, such a wide intrinsic bandwidth would neither be necessary nor desirable, since it would allow unnecessary out-of-band noise to propagate to subsequent stages. In a practical implementation, the signal bandwidth would therefore be restricted by dedicated downstream filtering. Alternatively, the intrinsic bandwidth of the preamplifier itself could be reduced through circuit-level optimization.

Reducing the intrinsic bandwidth of the preamplifier is not a neutral modification, since the same design parameters that control the dominant poles also affect transistor transconductance, parasitic capacitances, bias current, device noise, and the relative transfer functions of the main and auxiliary paths. Therefore, bandwidth reduction must be jointly optimized with noise, power consumption, gain, and cancellation accuracy rather than treated as an independent design variable.

### 4.3. Noise Performance

**Figure 7:**
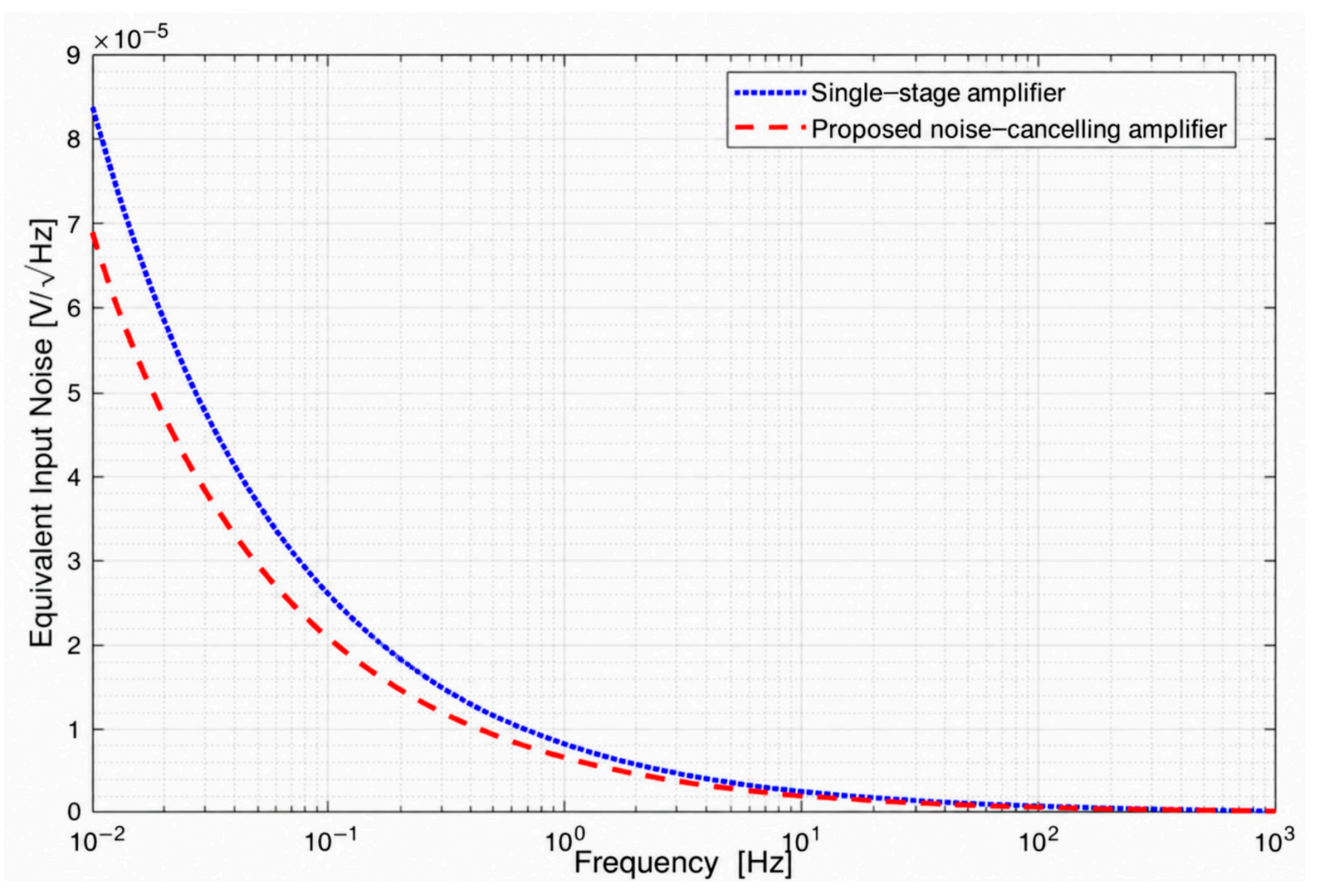
Simulated equivalent input noise spectral density of the reference single-stage amplifier and the proposed noise-cancelling amplifier.

The effectiveness of the proposed architecture was evaluated by comparing the equivalent input noise (EIN) and equivalent output noise (EON) of the reference single-stage amplifier and the proposed noise-cancelling topology over the frequency range of interest for biomedical signal acquisition. Figure 7 reports the simulated equivalent input noise spectral densities of the two amplifier configurations.

Although the two amplifier configurations exhibit different high-frequency bandwidths, this difference does not significantly affect the low-frequency noise comparison reported here. The −3 dB frequencies of the reference and proposed amplifiers are approximately 398.11 MHz and 251.89 MHz, respectively, whereas the noise results are evaluated over the 0.01 Hz–1 kHz interval. Therefore, throughout the entire noise-integration band, both circuits operate essentially within their flat mid-band region, more than five orders of magnitude below their respective cutoff frequencies.

The 1 kHz upper integration limit was selected as a system-level evaluation bandwidth representative of a subsequent bandwidth-limiting stage in a complete ECG acquisition front-end, rather than as a bandwidth imposed by the amplifier core itself. The investigated circuit intentionally does not include such a filtering stage, since its purpose is to isolate and evaluate the correlated noise-reduction mechanism at the preamplifier-core level. Accordingly, the reported integrated EIN and EON values represent noise integrated over the selected 0.01 Hz–1 kHz evaluation band and should not be interpreted as the total broadband noise of the uncompensated amplifier cores. The same bandwidth convention is adopted in the NEF calculation to ensure a consistent comparison between the reference and proposed architectures.

Consequently, the lower noise spectral density observed at 0.1 Hz, 1 Hz, and 10 Hz cannot be attributed to the different high-frequency bandwidths of the two architectures.

**Figure 8:**
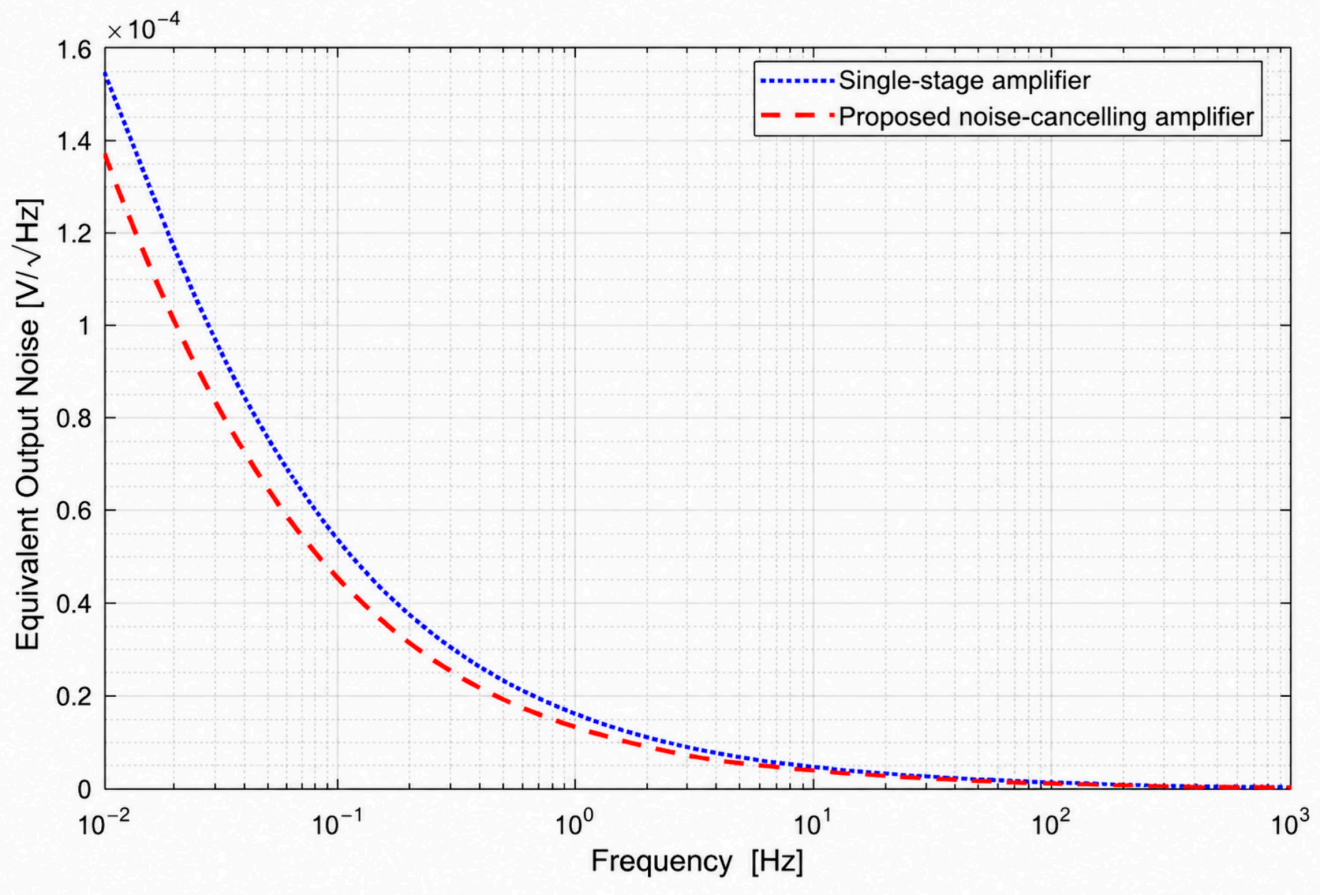
Simulated equivalent output noise spectral density of the reference single-stage amplifier and the proposed noise-cancelling amplifier.

Over the investigated frequency range, the proposed architecture exhibits a lower input-referred noise than the conventional single-stage amplifier, confirming that the auxiliary signal path effectively reduces the contribution of the internally generated noise. Similarly, Figure 8 compares the equivalent output noise spectra. Also in this case, the proposed architecture provides a measurable reduction of the output-referred noise over the entire investigated frequency range. This confirms that the observed improvement is not solely attributable to the increased voltage gain introduced by the auxiliary stage, but also reflects an actual reduction in the amplifier-generated noise. To provide a quantitative comparison within the selected ECG-oriented evaluation bandwidth, Table 1 summarizes the equivalent input- and output-noise spectral densities at representative frequencies together with the noise integrated over 0.01 Hz–1 kHz. The proposed architecture reduces the equivalent input noise by approximately 21.5% at 0.1 Hz and by about 19.9% at both 1 Hz and 10 Hz. Similarly, the output-referred noise is reduced by 14.1% at 0.1 Hz, 14.0% at 1 Hz, and 14.1% at 10 Hz. The integrated noise values further confirm this trend, showing a reduction of approximately 19.75% for the input-referred noise and 14% for the output-referred noise. The simulated spectra clearly indicate that both amplifiers operate in a flicker-noise-dominated regime at low frequencies. As expected for MOS transistors biased in weak inversion, the 1/f component largely exceeds the thermal contribution in the low-frequency range relevant to ECG acquisition. Consequently, the achievable noise reduction is primarily governed by the capability of the auxiliary path to attenuate the dominant low-frequency noise contribution generated by the input transistor.

The correlation assumption introduced in Section 3.3 was also quantitatively verified from the transistor-level small-signal noise transfer. Over the 0.01 Hz–1 kHz range, the extracted ratio |H_n,a_/H_n,b_| remained close to the first-order prediction α = 4.998 × 10^-4^ with values ranging from 4.96 × 10^-4^ at 0.1 Hz to 5.10 × 10^-4^ at 1 kHz. The corresponding phase difference remained below approximately 1.4° over the same frequency range and below 0.12° up to 10 Hz. When only the M_1_-generated contribution was considered, the magnitude-squared coherence between the noise components observed at V_a_ and V_b_ remained higher than 0.995 over the complete investigated range and higher than 0.998 in the low-frequency region up to 10 Hz. When all device-noise sources were included, the total-node coherence decreased moderately, from approximately 0.982 at 0.1 Hz to 0.964 at 10 Hz and 0.908 at 1 kHz, due to the increasing relative contribution of statistically independent noise sources. These results quantitatively confirm that, in the low-frequency flicker-dominated region relevant to the proposed methodology, the noise components sensed at V_a_ and V_b_ are predominantly correlated and originate from the same dominant M_1_ noise source.

The larger coherence observed at low frequency is also consistent with the flicker-noise-dominated operating regime of the circuit. Since M_1_ represents the dominant noise source in this region, the relative contribution of uncorrelated device-noise components is reduced, allowing the two internal noise observations to retain a stronger common-source correlation. At higher frequencies, the increasing relative weight of independent thermal and auxiliary-device noise contributions progressively reduces the total-node coherence.

**Figure 9:**
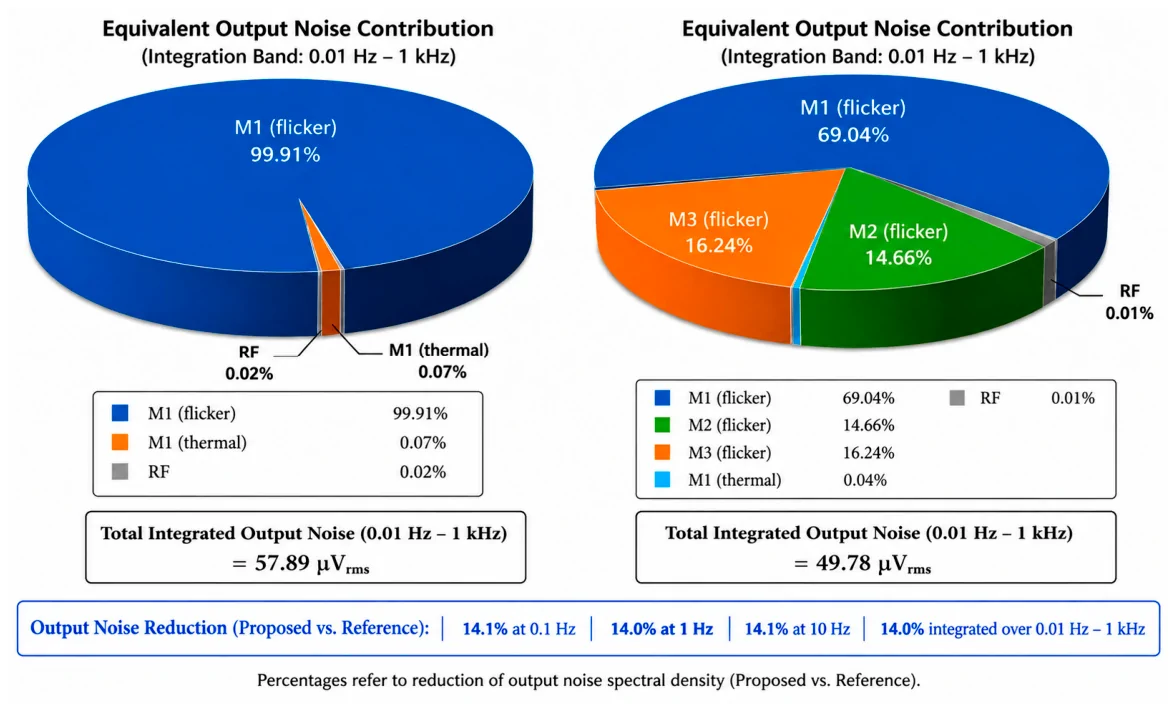
Summarized pie chart of the individual noise contribution of the main circuit components to the total equivalent output noise.

To further investigate the origin of the observed noise reduction, a noise-contribution analysis was performed by evaluating the individual contribution of each circuit component to the total output-referred noise. The individual contributions are combined in a root-sum-square sense because the corresponding device noise sources are statistically uncorrelated. The results are reported in Table 2 and summarized in Figure 9. For the reference topology, the root-sum-square of the individual contributions is approximately 58.0 µVrms, whereas for the proposed topology it is approximately 49.8 µVrms, consistent with the total output-referred noise reported in Table 1.

As expected, the single-stage amplifier is almost entirely dominated by the flicker noise generated by the input transistor M_1_, which accounts for approximately 99.9% of the total output-referred noise variance. The thermal noise contributions of both the transistor and the feedback resistor are more than three orders of magnitude lower and therefore have a negligible impact on the overall noise performance.

After introducing the auxiliary noise-reduction stage, the contribution of the flicker noise generated by M_1_ decreases significantly, from 99.9% to approximately 69% of the total noise. This reduction, together with the cross-spectral and coherence analysis reported above, confirms that the auxiliary branch attenuates the dominant M_1_-generated noise through the intended correlated-noise recombination mechanism.

At the same time, the auxiliary transistors M_2_ and M_3_ introduce their own flicker noise, contributing approximately 14.7% and 16.2%, respectively. Consequently, while the proposed architecture successfully reduces the dominant contribution of the input transistor, the overall improvement becomes limited by the intrinsic noise introduced by the auxiliary reduction stage itself.

This conclusion is further supported by the device-specific noise contribution analysis. In the reference topology, the noise contribution is almost entirely dominated by the flicker noise generated by M_1_, whereas in the proposed architecture the contribution associated with M_1_ is substantially reduced. Since this analysis isolates the contribution of the individual noise sources rather than relying only on the total integrated output noise, it provides direct evidence that the observed improvement originates from the auxiliary correlated-noise-reduction mechanism and not from high-frequency bandwidth limitation.

These results clearly highlight the fundamental design trade-off of the proposed architecture. Increasing the complexity of the noise-reduction network effectively reduces the dominant input-transistor noise but simultaneously introduces additional noise sources that establish a new lower bound for the achievable equivalent input noise.

The M_1_ flicker-noise contribution decreases from approximately 57.87 µVrms to 41.36 µVrms, corresponding to a 28.6% reduction. This result is in close agreement with the 28.6% attenuation predicted by the first-order model in Section 3.3, providing an independent transistor-level verification of the adopted partial noise-cancellation operating point.

Since the proposed topology exhibits a slightly higher voltage gain than the reference amplifier, the influence of this gain difference on the input-referred noise was also quantified. The mid-band gains of 6.34 dB and 6.95 dB correspond to linear gains of approximately 2.075 and 2.226, respectively, i.e., a gain ratio of 1.073. If the output-generated noise were unchanged, this gain increase alone would produce an apparent reduction of approximately 6.8% in the input-referred noise. Starting from the integrated EIN of the reference amplifier, this would correspond to approximately 26.1 µVrms. The simulated integrated EIN of the proposed topology is instead 22.49 µVrms, corresponding to an overall reduction of 19.75%. Therefore, only part of the observed EIN improvement can be attributed to the increased voltage gain.

This conclusion is independently confirmed by the output-referred noise, which decreases from 57.89 µVrms to 49.78 µVrms corresponding to approximately 14%. Since this quantity is evaluated directly at the output, its reduction demonstrates a genuine decrease in the amplifier-generated noise rather than an artifact of the increased voltage gain.

### 4.4. Statistical Robustness Analysis

To further evaluate the robustness of the proposed correlated noise-reduction technique, both Monte Carlo and Process–Voltage–Temperature (PVT) analyses were performed. While the Monte Carlo analysis assesses the sensitivity of the proposed architecture to statistical device mismatch, the PVT analysis investigates its behavior under representative operating conditions resulting from process, supply voltage, and temperature variations.

**Figure 10:**
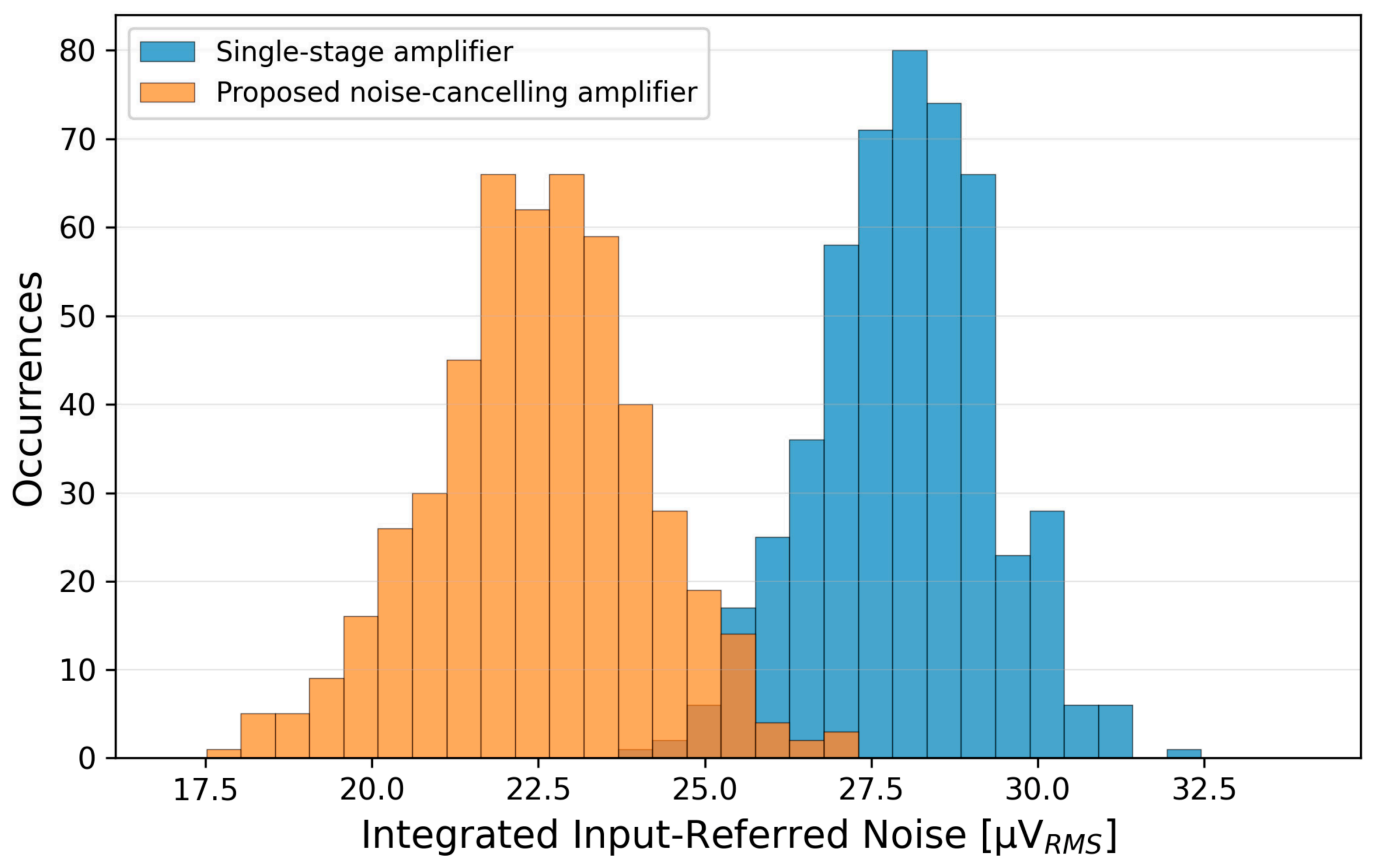
Monte Carlo distribution (500 runs) of the integrated input-referred noise for the conventional single-stage amplifier and the proposed noise-cancelling amplifier.

A Monte Carlo analysis consisting of 500 simulation runs was first carried out considering both process and mismatch variations. The integrated equivalent input noise was selected as the primary performance metric, since it directly quantifies the total amplifier-generated noise over the investigated frequency band and therefore represents the most meaningful figure of merit for low-frequency biomedical front-end amplifiers. The statistical distribution of the integrated input-referred noise obtained for both amplifier topologies is reported in Figure 10. The proposed noise-cancelling architecture consistently exhibits lower integrated noise values than the conventional single-stage amplifier throughout the entire Monte Carlo population. The mean integrated input-referred noise decreases from 28.00 µVrms for the reference topology to 22.51 µVrms for the proposed architecture, corresponding to an average reduction of approximately 19.6%, in excellent agreement with the nominal simulation results reported in Table 1. As expected, the proposed amplifier exhibits a slightly larger statistical dispersion than the reference implementation due to the additional auxiliary transistors M_2_ and M_3_, whose mismatch contributes to the overall variability of the circuit. Nevertheless, the increase in standard deviation remains limited, while the entire statistical distribution remains shifted toward lower integrated noise values.

This demonstrates that the proposed architecture retains its noise-reduction capability despite the additional active devices required to realize the correlated noise-reduction mechanism.

**Figure 11:**
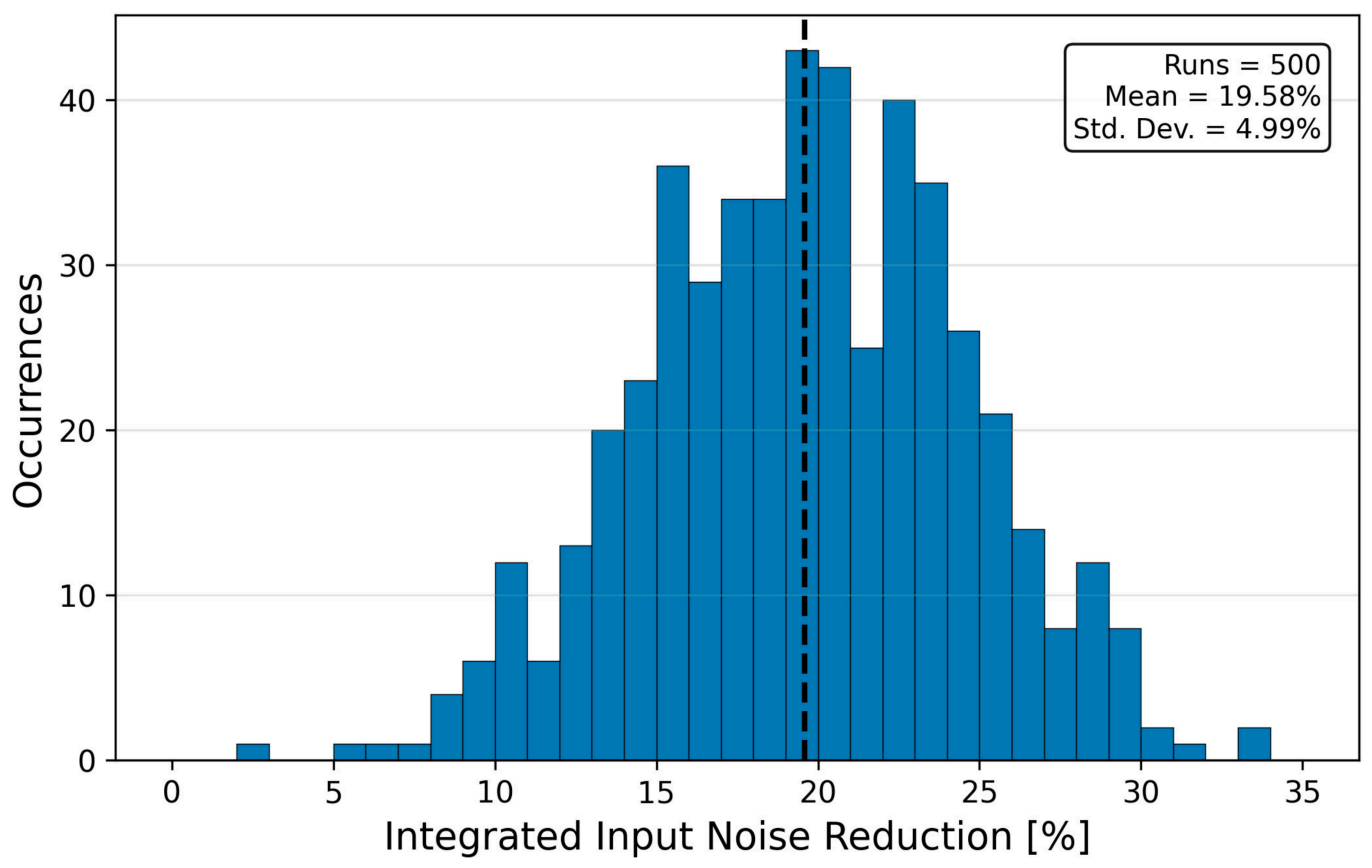
Monte Carlo distribution (500 runs) of the integrated input-referred noise reduction achieved by the proposed noise-cancelling amplifier with respect to the reference architecture.

To further quantify the consistency of the proposed approach, Figure 11 reports the statistical distribution of the percentage reduction of the integrated input-referred noise with respect to the conventional amplifier. The obtained distribution is centered around a mean improvement of approximately 19.6%, indicating that the proposed architecture consistently reduces the amplifier-generated noise across the entire Monte Carlo population. No simulated sample exhibits a degradation in performance with respect to the reference topology, confirming that the correlated noise-reduction mechanism remains effective under statistically significant manufacturing variations.

**Figure 12:**
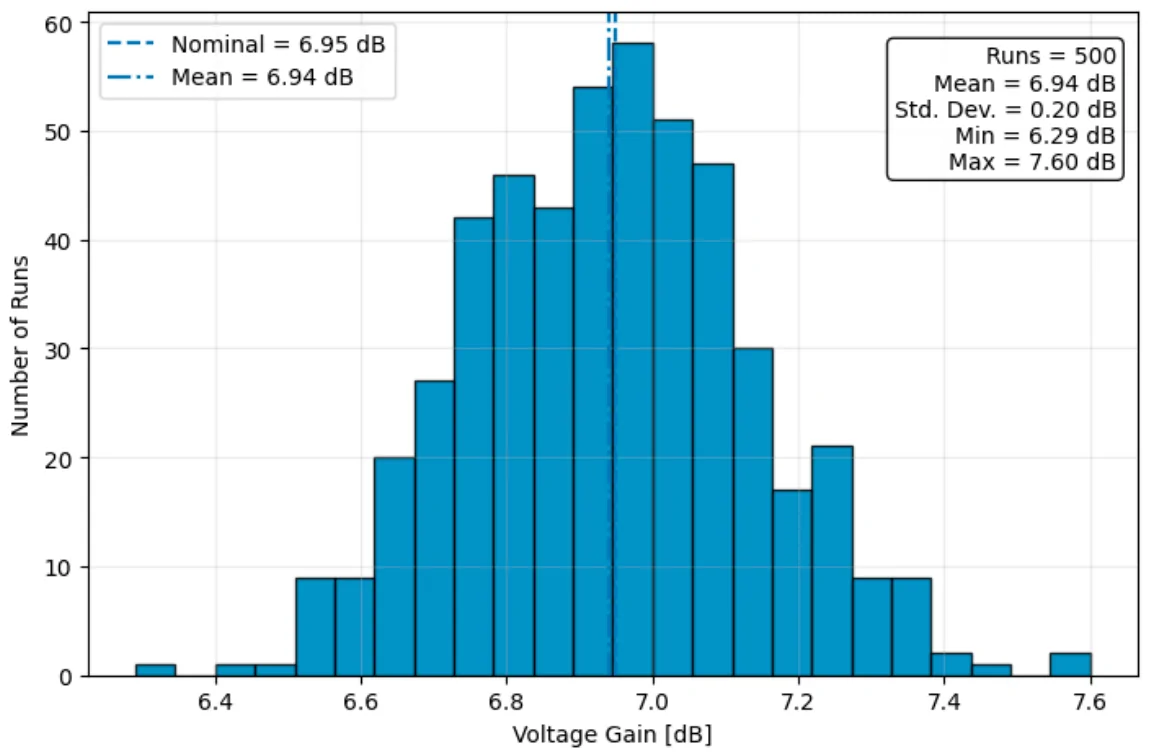
Monte Carlo distribution (500 runs) of the voltage gain of the proposed noise-cancelling preamplifier.

To further verify the functional robustness of the proposed architecture, the voltage gain was also monitored over the same 500 Monte Carlo runs. As shown in Figure 12, the gain exhibits a mean value of 6.94 dB with a standard deviation of 0.20 dB, remaining close to the nominal value of 6.95 dB. The observed spread is limited, with gain values ranging from approximately 6.29 dB to 7.60 dB, confirming that the amplifier preserves its amplification capability under process and mismatch variations.

**Figure 13:**
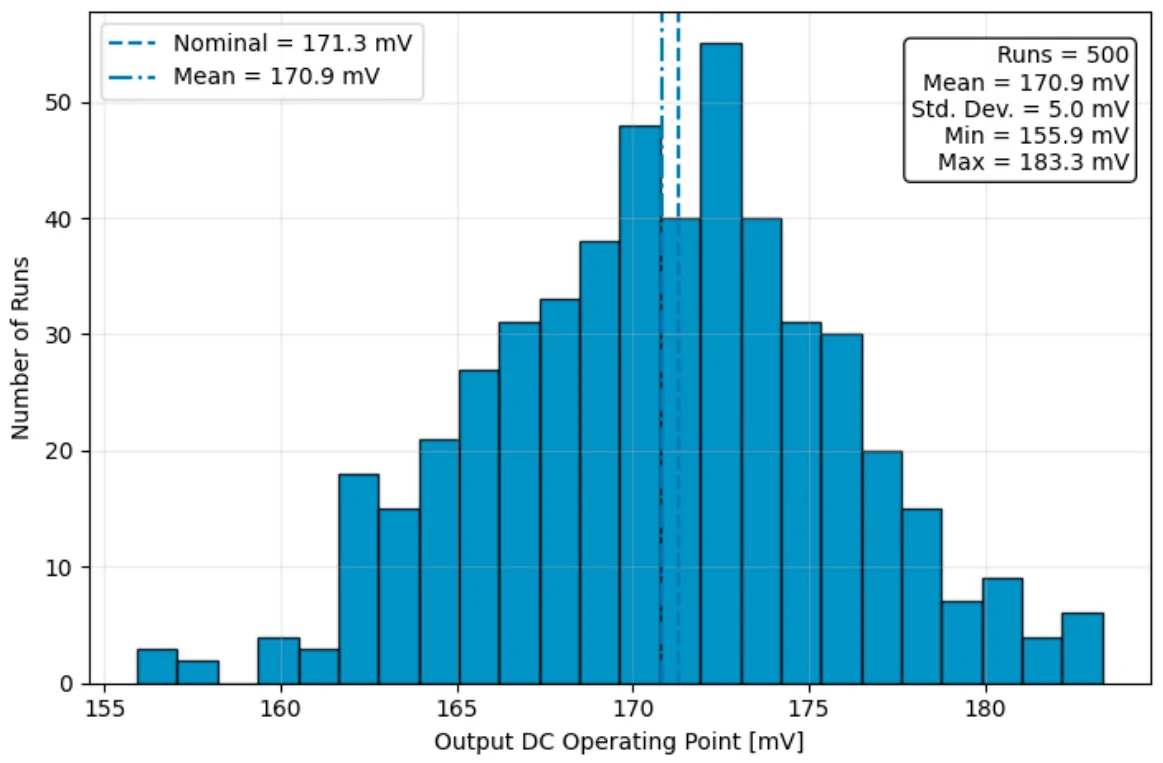
Monte Carlo distribution of the output DC operating point of the proposed noise-cancelling amplifier over 500 runs.

The DC operating point of the output branch was also monitored over the same 500 Monte Carlo runs, reported in Figure 13. The resulting distribution exhibits a mean value of 170.9 mV and a standard deviation of 5.0 mV, compared with the nominal value of 171.3 mV. Across the entire statistical population, the output DC voltage remains between approximately 155.9 mV and 183.3 mV. This limited spread confirms that the output branch remains properly biased and that no loss of functionality is observed under the considered process and mismatch variations.

In addition to the statistical analysis, the proposed amplifier was evaluated under representative PVT corners in order to assess the robustness of the proposed architecture under different operating conditions. The results are summarized in Table 3. As expected, the SS corner represents the most critical operating condition due to the reduced transistor transconductance and the increased contribution of low-frequency noise, leading to the highest integrated equivalent input noise. Conversely, the FF corner provides the lowest integrated noise owing to the increased carrier mobility and transconductance. Despite these variations, the proposed architecture maintains a consistent reduction of the integrated input-referred noise across all investigated operating conditions. The resulting improvement ranges from approximately 17% under the worst-case SS corner to more than 22% under the FF corner, demonstrating that the effectiveness of the correlated noise-reduction mechanism is preserved independently of process, voltage, and temperature variations.

The PVT results show that the auxiliary transconductances remain of the same order of magnitude as their nominal values, while the proposed topology preserves a positive noise-reduction margin over all investigated corners.

Overall, the combined Monte Carlo and PVT analyses demonstrate that the proposed amplifier does not rely on a single nominal operating point, but retains its noise-reduction capability under both statistical mismatch and environmental variations. These results further support the applicability of the proposed architecture to practical low-noise biomedical front-end interfaces, where robustness to manufacturing tolerances and operating conditions is a fundamental design requirement.

To complement the analytical discussion presented in Section 3.3, the sensitivity of the proposed noise-reduction mechanism to the equivalent source resistance was further investigated through transistor-level noise simulations. The source resistance $R_{S}$ was swept over representative values of 50 Ω, 1 kΩ, 10 kΩ, and 100 kΩ, while all remaining circuit parameters and bias conditions were kept unchanged; the results have been reported in Table 4. For each value of $R_{S}$, the integrated input-referred noise over the 0.01 Hz–1 kHz interval was evaluated for both the reference and proposed architectures. In addition, the M_1_-generated noise contribution was monitored separately in order to directly assess the effectiveness of the correlated-noise-reduction mechanism.

The results confirm that the proposed architecture maintains a positive noise-reduction margin over the complete investigated source-resistance range. Starting from the nominal $R_{S}=$ 50 Ω condition, for which the integrated EIN reduction is approximately 19.75%, the improvement progressively increases with $R_{S}$, reaching approximately 29.1% at $R_{S}=100\;$kΩ. A similar trend is observed for the M_1_-specific noise contribution, whose attenuation increases from approximately 28.6% to 53.5%. This behavior is consistent with the first-order analysis of Section 3.3, since increasing $R_{S}$ reduces the auxiliary transconductance required for ideal cancellation and therefore moves the implemented operating point closer to the ideal cancellation condition.

At the highest source-resistance value, $R_{S}=100\;k\Omega$, the transistor-level M_1_-noise attenuation remains significantly larger than under the nominal 50 Ω condition, although it is lower than the value predicted by the first-order model. This deviation is expected because $R_{S}$ becomes comparable with $R_{F}=100\;k\Omega$, making the simplifying assumptions adopted in the analytical derivation progressively less accurate. In particular, finite device output conductances, changes in the effective node impedances, loading effects, and the frequency dependence of the internal noise-transfer functions become more relevant. Therefore, the analytical model correctly predicts the improvement trend with increasing $R_{S}$, while the transistor-level simulations provide a more conservative estimate of the achievable cancellation.

It should also be noted that the improvement in the total integrated EIN remains smaller than the reduction of the M_1_-specific contribution because the residual noise floor is progressively limited by the intrinsic noise generated by the auxiliary devices M_2_ and M_3_. Overall, these results demonstrate that the proposed noise-reduction mechanism does not rely exclusively on the nominal 50 Ω source condition and remains effective over a substantially wider source-resistance range. Nevertheless, a practical ECG electrode exhibits a complex and frequency-dependent impedance; therefore, validation using a complete electrode–skin interface model is left to future system-level investigation.

### 4.5. ECG Signal Validation

The practical impact of the proposed noise-reduction architecture was further evaluated through a time-domain simulation using a representative ECG waveform. In this analysis, the proposed circuit was considered an amplification block embedded within a more complete analog front-end, rather than as a standalone ECG denoising stage. The objective was therefore not to demonstrate the removal of noise already present in the acquired ECG signal, but to assess how the reduction of the noise internally generated by the amplification stage affects the quality of the waveform delivered to the subsequent blocks of the acquisition chain.

A representative ECG waveform with a peak amplitude of approximately 1 mV was adopted to evaluate the effect of the proposed noise-reduction architecture under a controlled time-domain excitation. To reproduce a non-ideal signal delivered by a preceding electrode-conditioning stage, the clean ECG waveform was intentionally corrupted by two common input disturbances. First, a zero-mean band-limited Gaussian noise component with an RMS amplitude of 150 µV was generated over the 0.5–100 Hz frequency range. Second, baseline wander was modeled as a sinusoidal component with a peak amplitude of 200 µV and a frequency of 0.25 Hz. The resulting common input waveform can therefore be expressed as:

(19) $$v_{in}\left(t\right)=v_{ECG}\left(t\right)+v_{n,ext}\left(t\right)+v_{BW}\left(t\right)$$

where *v_ECG_*(*t*) is the clean ECG waveform, *v_n,ext_*(*t*) is the band-limited external noise, and

(20) $$v_{BW}\left(t\right)=200\;\mu V\sin\left(2\pi \cdot 0.25\;Hz\cdot t\right)$$

represents the imposed baseline-wander component. The same time-domain realization of *v_n,ext_*(*t*) and *v_BW_*(*t*) was applied to both amplifier configurations. Therefore, any difference observed between the two output waveforms is not caused by different input-noise realizations.

The intrinsic noise generated by the two amplifier architectures was treated separately from the common input disturbances. For the time-domain comparison, output-referred stochastic noise waveforms were generated with a flicker-noise-dominated spectral profile and a maximum noise frequency of 1 kHz, consistent with the integration bandwidth adopted in Section 4.3. Their RMS amplitudes were normalized to the integrated output-referred noise values obtained from the transistor-level noise analysis, namely 57.89 µV_rms_ for the reference single-stage amplifier and 49.78 µV_rms_ for the proposed architecture. These values correspond to the same 0.01 Hz–1 kHz integration interval used in Table 1. The resulting output waveforms can therefore be represented as

(21) $$v_{out,ref}\left(t\right)=A_{ref}v_{in}\left(t\right)+v_{n,ref}\left(t\right)$$

and

(22) $$v_{out,prop}\left(t\right)=A_{prop}v_{in}\left(t\right)+v_{n,prop}\left(t\right)$$

where *A_ref_* and *A_prop_* are the corresponding linear voltage gains. The simulated mid-band gains of 6.34 dB and 6.95 dB correspond to 2.075 *V*/*V* and 2.226 *V*/*V*, respectively.

**Figure 14:**
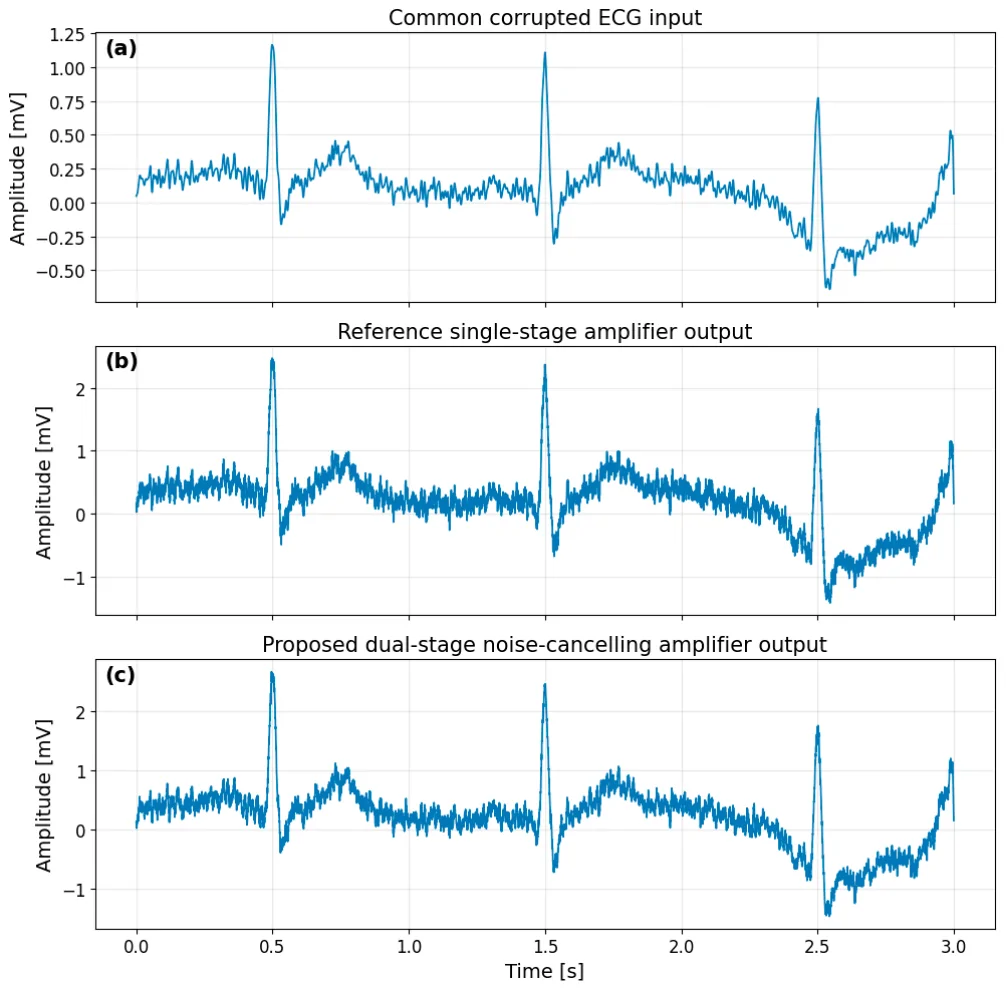
Time-domain comparison of two alternative amplification paths under the same noisy ECG excitation. (**a**) Noisy ECG input stimulus applied to both front-ends. (**b**) Output of the reference single-stage amplifier without noise reduction. (**c**) Output of the proposed noise-cancelling amplifier.

Figure 14 shows the resulting transient waveforms. Figure 14a reports the common corrupted ECG waveform applied identically to both amplifier configurations, whereas Figure 14b and Figure 14c show the outputs of the reference single-stage amplifier and the proposed noise-cancelling amplifier, respectively. The two output plots are reported using consistent time and amplitude scales in order to avoid an artificial visual enhancement of the difference between the two architectures.

Because the externally added noise and baseline wander are identical for the two circuits and are not targeted by the proposed cancellation mechanism, the time-domain comparison was complemented by a quantitative evaluation of the degradation introduced specifically by the amplifier itself. For this purpose, an amplifier-related output signal-to-noise ratio was calculated as

(23) $$SNR_{amp}=20\log_{10}\frac{V_{ECG,out,rms}}{V_{n,amp,rms}}$$

where *V_ECG,out,rms_* is the RMS value of the amplified clean ECG component and *V_n,amp,rms_* is the RMS internally generated output noise of the corresponding amplifier. Under the adopted conditions, the reference amplifier yields an amplifier-related output SNR of 15.43 dB, whereas the proposed topology achieves 17.35 dB, corresponding to an improvement of 1.92 dB.

When the externally imposed noise and baseline-wander components are also included in the SNR calculation, the improvement is necessarily much smaller because these disturbances are common to both amplifier inputs and are amplified together with the useful ECG signal. This distinction is important because the proposed architecture is not intended to remove interference already present at its input. Instead, its purpose is to reduce the additional noise generated internally by the amplification stage itself.

Accordingly, the proposed topology should not be interpreted as an alternative to conventional ECG denoising techniques such as analog or digital filtering, adaptive filtering, or baseline-wander suppression. Those techniques act on disturbances already superimposed on the acquired biopotential signal, whereas the present architecture addresses the internally generated noise of the amplification core. The lower output-referred noise therefore limits the additional degradation introduced by the first active stage and improves preservation of the ECG waveform before subsequent filtering, programmable gain, and analog-to-digital conversion.

The results of Figure 14 thus provide a quantitative system-level illustration of the circuit-level noise analysis presented in Section 4.3. In particular, the transient comparison is consistent with the reduction of the integrated output-referred noise from 57.89 µV_rms_ to 49.78 µV_rms_. Rather than suggesting removal of the externally applied noise, the results demonstrate that the correlated noise-reduction path allows the ECG waveform to be amplified while introducing a smaller additional internally generated noise contribution than the reference topology.

A direct quantitative comparison with prior ECG front-end amplifiers is not straightforward, because the proposed work does not target a complete ECG analog front-end but rather a specific noise-cancellation methodology at the preamplifier-core level. Most state-of-the-art biomedical front-ends reduce low-frequency noise through chopper stabilization, auto-zeroing, impedance-boosting techniques, or complete instrumentation-amplifier architectures, whereas correlated noise cancellation has been mainly developed in RF and wideband LNAs. As a result, no directly comparable state-of-the-art circuit class combines low-frequency ECG-oriented operation, flicker-noise-dominated CMOS amplification, and RF-inspired correlated internal noise cancellation.

For this reason, the comparison is mainly performed against a reference single-stage amplifier implemented under the same technology, biasing conditions, and input-stage sizing. This comparison allows the isolated contribution of the proposed cancellation path to be evaluated without introducing differences in complete front-end architecture, filtering strategy, electrode interface, or system-level gain distribution.

## 5. Conclusions

This work presented a methodology for applying correlated noise-reduction principles, originally developed for wideband and RF low-noise amplifiers, to low-frequency biomedical front-end circuits operating in a flicker-noise-dominated regime. Rather than targeting the complete cancellation of the noise generated by the input transistor, the proposed approach introduces an auxiliary feedforward path designed to reduce its contribution while preserving the useful signal and maintaining practical circuit complexity.

The methodology was applied to a resistive-feedback common-source amplifier implemented in a 150 nm CMOS technology and was evaluated through small-signal, noise, Monte Carlo, PVT, and time-domain simulations. The noise-contribution analysis confirmed that the auxiliary path attenuates the dominant flicker-noise contribution of the input transistor, although the overall improvement is ultimately limited by the noise introduced by the additional devices.

The statistical analyses further showed that the noise-reduction mechanism remains effective under process, mismatch, supply-voltage, and temperature variations. Moreover, the ECG time-domain analysis demonstrated that, when the circuit is embedded as an amplification block within a more complete analog front-end, the reduction of internally generated noise results in lower additional degradation of the biomedical waveform. This result should not be interpreted as the removal of noise already present at the input, but as improved signal preservation during the amplification process.

The proposed circuit should therefore be regarded primarily as a proof-of-concept implementation of a broader design methodology rather than as a complete ECG analog front-end. The main contribution of this work lies in demonstrating how correlated internal noise components can be identified, processed, and partially recombined to improve the noise performance of low-frequency CMOS amplifiers under practical transconductance, device-sizing, and circuit-complexity constraints. Future developments will focus on the optimization of the auxiliary branch in terms of noise, power consumption, and area, integrating the proposed core into a complete differential biomedical front-end, and performing experimental validation through integrated-circuit measurements.

## Figures and Tables

**Table 1:** Equivalent input and output noise comparison between the reference single-stage amplifier and the proposed noise-cancelling amplifier.

Parameter	Single-Stage	Proposed Dual Stage	Improvement (%)
EIN @0.1 Hz [μV/√Hz]	26.097	20.486	21.5
EIN @1 Hz [μV/√Hz]	8.251	6.614	19.9
EIN @10 Hz [μV/√Hz]	2.609	2.092	19.9
Integrated EIN (0.01 Hz to 1 kHz) [μV_rms_]	28.03	22.49	19.75
EON @0.1 Hz [μV/√Hz]	54.10	46.49	14.1
EON @1 Hz [μV/√Hz]	17.12	14.72	14.0
EON @10 Hz [μV/√Hz]	5.418	4.653	14.1
Integrated EON (0.01 Hz to 1 kHz) [μV_rms_]	57.89	49.78	14.0

**Table 2:** Individual integrated output-referred noise contributions of the main circuit components over the 0.01 Hz–1 kHz frequency range.

Noise Source	Single-Stage [μVrms]	Noise-Variance Contribution [%]	Proposed Dual-Stage [μVrms]	Noise-Variance Contribution [%]
M1 (flicker)	57.866	99.91	41.36	69.04
M1 (thermal)	1.41	0.06	0.996	0.04
R_F_ thermal (100 kΩ)	0.819	0.02	0.498	0.01
R_L_ thermal (200 kΩ)	0.25	0.002	0.18	0.0013
M2 (flicker)	-	-	19.06	14.66
M3 (flicker)	-	-	20.06	16.24

**Table 3:** PVT analysis of the proposed noise-cancelling amplifier.

Corner	VDD, M1 [mV]	VDD, M2-M3 [mV]	g_m1_ [µS]	g_m2_ [mS]	g_m3_ [mS]	Gain [dB]	Single-Stage EIN [µV_rms_]	Proposed EIN [µV_rms_]	Improvement [%]
TT, 27 °C	460	400	46.7	2.13	2.17	6.95	28.03	22.49	19.75
SS, 85 °C	430	374	37.9	1.72	1.76	6.1	30.20	25.10	16.9
FF, −20 °C	490	426	57.4	2.78	2.82	7.4	26.87	20.85	22.4
SF, 27 °C	430	374	42.0	1.95	2.34	6.4	29.24	23.80	18.6
FS, 27 °C	490	426	51.4	2.42	2.08	7.1	27.75	21.95	20.9

Note: The two supply rails were varied simultaneously while preserving the same relative deviation from their nominal values. The nominal supply voltages are 460 mV for the M_1_ input stage and 400 mV for the M_2_–M_3_ auxiliary branch.

**Table 4:** Source-resistance sensitivity of the integrated input-referred noise over the 0.01 Hz–1 kHz frequency range.

Rs	Reference EIN [µVrms]	Proposed EIN [µVrms]	EIN Reduction [%]	M1-Noise Reduction [%]
50 Ω	28.03	22.49	19.75	28.6
1 kΩ	28.4	22.55	20.6	29.2
10 kΩ	30.1	22.8	24.3	34.7
100 kΩ	38.5	27.3	29.1	53.5

## Data Availability

The original contributions presented in this study are included in the article. Further inquiries can be directed to the corresponding author.

## References

[1] Olivieri R., Ferri G. (2026). Recent Advances in Analog Front-End Architectures for ECG Signal Acquisition. IEEE Sens. Rev..

[2] Omran A., Aprile A., Moisello E., Tatu V., Calabró R., Malcovati P., Bonizzoni E. (2025). Toward a Generalized Analog Front End for Multiple Biomedical Signals Acquisition: A Review. IEEE Sens. J..

[3] Ranjbar Koleibi E., Lemaire W., Koua K., Benhouria M., Bostani R., Serri Mazandarani M., Gauthier L.-P., Besrour M., Ménard J., Majdoub M. (2025). Design and Implementation of a Low-Power Biopotential Amplifier in 28 nm CMOS Technology with a Compact Die-Area of 2500 μm^2^ and an Ultra-High Input Impedance. Sensors.

[4] Emon M.Z.A., Salim K.M., Chowdhury M.I.B. (2024). Design and Analysis of a High-Gain, Low-Noise, and Low-Power Analog Front End for Electrocardiogram Acquisition in 45 nm Technology Using gm/ID Method. Electronics.

[5] Hall D.A., Makinwa K.A.A., Jang T. (2023). Quantifying Biomedical Amplifier Efficiency: The Noise Efficiency Factor. IEEE Solid-State Circuits Mag..

[6] Enz C.C., Temes G.C. (1996). Circuit Techniques for Reducing the Effects of Op-Amp Imperfections: Autozeroing, Correlated Double Sampling, and Chopper Stabilization. Proc. IEEE.

[7] Chan P.K., Ng K.A., Zhang X.L. A CMOS Chopper-Stabilized Differential Difference Amplifier for Biomedical Integrated Circuits. Proceedings of the 47th IEEE Midwest Symposium on Circuits and Systems (MWSCAS 2004).

[8] Blaakmeer S.C., Klumperink E.A.M., Leenaerts D.M.W., Nauta B. (2008). Wideband Balun-LNA With Simultaneous Output Balancing, Noise-Canceling and Distortion-Canceling. IEEE J. Solid-State Circuits.

[9] Razavi B. (2012). RF Microelectronics.

[10] Kim J., Hoyos S., Silva-Martinez J. (2010). Wideband Common-Gate CMOS LNA Employing Dual Negative Feedback with Simultaneous Noise, Gain, and Bandwidth Optimization. IEEE Trans. Microw. Theory Tech..

[11] Khanehbeygi M., Jafarnejad R., Koozehkanani Z.D., Sobhi J. (2022). A Low-Power Wideband LNA Exploiting Current-Reuse and Noise-Cancellation Techniques. Int. J. Circuit Theory Appl..

[12] Bruccoleri F., Klumperink E.A.M., Nauta B. (2004). Wide-Band CMOS Low-Noise Amplifier Exploiting Thermal Noise Canceling. IEEE J. Solid-State Circuits.

[13] Friis H.T. (1944). Noise Figures of Radio Receivers. Proc. IRE.

[14] Olivieri R., Barile G., Stornelli V., Zompanti A., Santonico M., Ferri G. A Low-Noise VCII for Biosignal Conditioning. Proceedings of the International Workshop on Biomedical Applications, Technologies and Sensors (BATS).

[15] Fang L., Gui P. (2022). A Low-Noise Low-Power Chopper Instrumentation Amplifier with Robust Technique for Mitigating Chopping Ripples. IEEE J. Solid-State Circuits.

[16] Huang Y.-K., Rodriguez S. (2024). Noise Analysis and Design Methodology of Chopper Amplifiers with Analog DC-Servo Loop for Biopotential Acquisition Applications. IEEE Trans. Very Large Scale Integr. Syst..

[17] Tsai J. (2025). Research and Design on Noise Performance Optimization of Operational Amplifiers. Appl. Comput. Eng..

[18] Xu W., Wang T., Wei X., Yue H., Wei B., Duan J., Li H. (2020). Low Noise, High Input Impedance Digital-Analog Hybrid Offset Suppression Amplifier for Wearable Dry Electrode ECG Monitoring. Electronics.

[19] Guermandi M., Scarselli E.F., Guerrieri R. (2016). A Driving Right Leg Circuit (DgRL) for Improved Common-Mode Rejection in Biopotential Acquisition Systems. IEEE Trans. Biomed. Circuits Syst..

